# Advances in establishment and analysis of three-dimensional tumor spheroid-based functional assays for target validation and drug evaluation

**DOI:** 10.1186/1741-7007-10-29

**Published:** 2012-03-22

**Authors:** Maria Vinci, Sharon Gowan, Frances Boxall, Lisa Patterson, Miriam Zimmermann, William Court, Cara Lomas, Marta Mendiola, David Hardisson, Suzanne A Eccles

**Affiliations:** 1 Cancer Research UK Cancer Therapeutics Unit, The Institute of Cancer Research, Sutton, SM2 5NG, UK; 2 Laboratory of Pathology and Oncology, Research Unit, Fundación para la Investigación Biomedica de Hospital Universitario La Paz (FIBHULP), IdiPAZ, 28046 Madrid, Spain; 3 Department of Pathology, Hospital Universitario La Paz, Universidad Autónoma de Madrid, IdiPAZ, 28046 Madrid, Spain

**Keywords:** 3D, angiogenesis, drug response, high throughput, invasion, migration, tumor spheroids

## Abstract

**Background:**

There is overwhelming evidence that *in vitro *three-dimensional tumor cell cultures more accurately reflect the complex *in vivo *microenvironment than simple two-dimensional cell monolayers, not least with respect to gene expression profiles, signaling pathway activity and drug sensitivity. However, most currently available three-dimensional techniques are time consuming and/or lack reproducibility; thus standardized and rapid protocols are urgently needed.

**Results:**

To address this requirement, we have developed a versatile toolkit of reproducible three-dimensional tumor spheroid models for dynamic, automated, quantitative imaging and analysis that are compatible with routine high-throughput preclinical studies. Not only do these microplate methods measure three-dimensional tumor growth, but they have also been significantly enhanced to facilitate a range of functional assays exemplifying additional key hallmarks of cancer, namely cell motility and matrix invasion. Moreover, mutual tissue invasion and angiogenesis is accommodated by coculturing tumor spheroids with murine embryoid bodies within which angiogenic differentiation occurs. Highly malignant human tumor cells were selected to exemplify therapeutic effects of three specific molecularly-targeted agents: PI-103 (phosphatidylinositol-3-kinase (PI3K)-mammalian target of rapamycin (mTOR) inhibitor), 17-*N*-allylamino-17-demethoxygeldanamycin (17-AAG) (heat shock protein 90 (HSP90) inhibitor) and CCT130234 (in-house phospholipase C (PLC)γ inhibitor). Fully automated analysis using a Celigo cytometer was validated for tumor spheroid growth and invasion against standard image analysis techniques, with excellent reproducibility and significantly increased throughput. In addition, we discovered key differential sensitivities to targeted agents between two-dimensional and three-dimensional cultures, and also demonstrated enhanced potency of some agents against cell migration/invasion compared with proliferation, suggesting their preferential utility in metastatic disease.

**Conclusions:**

We have established and validated a suite of highly reproducible tumor microplate three-dimensional functional assays to enhance the biological relevance of early preclinical cancer studies. We believe these assays will increase the translational predictive value of *in vitro *drug evaluation studies and reduce the need for *in vivo *studies by more effective triaging of compounds.

## Background

The preclinical validation process in cancer drug discovery generally comprises a series of primary biochemical and cell-based assays, followed by evaluation in animal tumor models. However, there is a high rate of attrition and fewer than 10% of candidates identified by high-throughput (HT) screening become licensed drugs [1]. Standard two-dimensional cell cultures for testing effects of anticancer agents are simple and convenient, but present significant limitations in reproducing the complexity and pathophysiology of *in vivo *tumor tissue [2-8].

Three-dimensional culture systems are of increasing interest in cancer research since tissue architecture and the extracellular matrix (ECM) significantly influence tumor cell responses to microenvironmental signals. For example, inhibition of β1-integrin on breast tumor cells reverted their morphological and functional features to a normal epithelial phenotype, but only in three dimensions [9]. This and many other studies have highlighted significant differences between two-dimensional and three-dimensional cultures, with the latter reflecting better the *in vivo *tumor microenvironment in terms of cellular heterogeneity, nutrient and oxygen gradients, cell-cell interactions, matrix deposition and gene expression profiles [5,6,8-11]. To model solid tumors more effectively, several three-dimensional culture systems have been established: whole perfused organs, tissue explants, scaffold/microcarrier-based cultures, hollow-fiber bioreactors, organotypic cultures (multicellular spheroids and cellular multilayers) and gel/matrix-based cultures [3,4,12-15]. Of these, the multicellular tumor spheroid model is the best characterized and most widely used.

Tumor spheroids are heterogeneous cellular aggregates that, when greater than 500 μm diameter, are frequently characterized by hypoxic regions and necrotic centers [16,17]. Three-dimensional spheroids are therefore considered valid models to recapitulate features of tumor microregions, intervascular domains or micrometastases [10,12]. Sutherland *et al. *first applied this technique in cancer research [18]. Since then, several methods have been used to generate tumor spheroids: spontaneous aggregation [19,20], spinner flasks [21], rotary cell culture systems [3,22], poly-2-hydroxyethyl methacrylate (poly-Hema)-coated plates [23,24], hanging drops [25], liquid overlay on agar [26-28], low binding plates [29,30], gel/matrix-based culture [31], polymeric scaffolds [11] or micropatterned plates [32,33].

Each method has advantages and limitations [3,13] but simple, standardized and rapid protocols appropriate for routine preclinical drug development studies within academic or pharmaceutical labs are lacking. Furthermore, there is an appreciation that the next generation of anticancer drugs will need to address aspects of the malignant phenotype beyond cell proliferation if we are to effectively address disseminated disease. We have therefore developed a complete suite of *in vitro *three-dimensional spheroid-based assays for measuring tumor growth, migration, invasion and tumor angiogenesis that, combined with new technologies of automated imaging and analysis, demonstrate applicability in relatively high-throughput formats.

We initially screened and classified a diverse collection of human tumor cell lines for their ability to form spheroids. For more detailed assay exemplification, selected lines representing highly malignant human tumors and characterized by an optimal three-dimensional structure were used. The following report is divided into two sections: a description of the establishment of the suite of three-dimensional spheroid growth and functional assays, and an exemplification of the utility of the assays to measure inhibitory effects of molecularly targeted agents. Inhibitors of heat shock protein 90 (HSP90) chaperone (17-*N*-allylamino-17-demethoxygeldanamycin (17-AAG)), phosphatidylinositol-3-kinase (PI3K)-mammalian target of rapamycin (mTOR) (PI-103) and phospholipase C (PLC)γ (CCT130234), representing a variety of validated targets in tumor growth, invasion and angiogenesis [34-37], were selected for evaluation. We believe that these assays will contribute to increased utility and predictive value of preclinical drug discovery studies.

## Results

### Establishment of three-dimensional spheroid-based assays

#### Rapid generation of reproducibly sized tumor spheroids and automated growth kinetic analyses

To deploy three-dimensional cultures in cancer drug discovery and target validation, we established a standardized microplate method with the following desirable characteristics: (i) 96-well suspension culture; (ii) a single spheroid/well, centered for ease of optical imaging; (iii) high reproducibility; (iv) simple harvesting for further analysis; (v) fully automated imaging and quantitative analysis.

**Figure 1:**
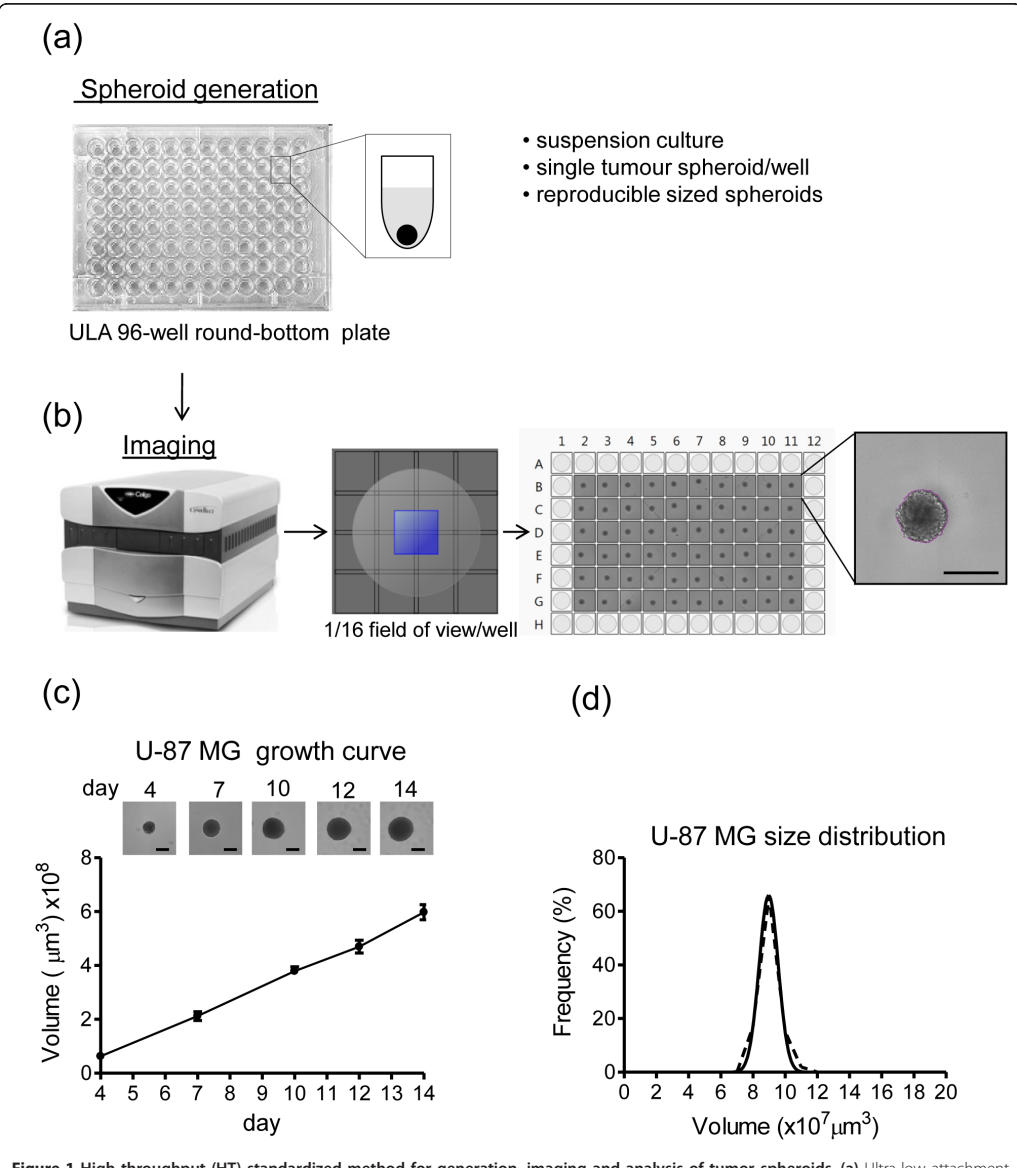
**High throughput (HT) standardized method for generation, imaging and analysis of tumor spheroids**. **(a) **Ultra-low attachment (ULA) 96-well round-bottomed plates were used to generate suspension cultures of reproducibly sized, single spheroids in each well. **(b) **Fully automated imaging and analysis was carried out on a Celigo cytometer using the Colony Counting Embryoid Body application with 1/16 field of view/well. A screen print shows reproducibly sized U-87 MG spheroids across the plate and detail of the segmentation analysis around the spheroid. Scale bar: 500 μm. **(c) **U-87 MG tumor spheroid growth curve. Values are means ± SD (n = 6). Representative images obtained on Celigo for each timepoint are shown. Scale bar 500 μm. **(d) **Frequency plots of 4-day U-87 MG spheroid volume (μm^3 ^× 10^7^) across a plate containing 60 spheroids.

We used ultra-low attachment (ULA) 96-well round-bottomed plates that, in contrast to standard methods, do not require coating to prevent cell adhesion. Tumor cell suspensions formed a three-dimensional structure within 24 to 48 h. The well shape promotes the formation of single, centrally located spheroids of reproducible size (Figure 1a). Optimal seeding densities were established such that tumor spheroids for every cell line tested (regardless of their proliferative potential and cell cycle time) fell within a size range of 300 to 500 μm in diameter on day 4, considered appropriate for initiating experimental studies. Cultures were maintained by replacing 50% of the medium on days 4, 7, 10 and 12.

For rapid, routine imaging and analysis of tumor spheroids, we utilized a Celigo™ cytometer (Cyntellect Inc, San Diego, CA, USA; http://www.cyntellect.com/content/products/celigo/index.html) (Figure 1b), which is a benchtop *in situ *cellular analysis system providing high quality, full or partial images of wells using brightfield or fluorescent illumination. Images were acquired and analyzed using the Colony Counting Embryoid Body (EB) application that identifies and counts individual three-dimensional structures (EBs or spheroids) and small clusters of cells. With our centrally located spheroids and by selecting 1/16 field of view/well (Figure 1b) the Celigo cytometer scans a ULA 96-well plate in 8 minutes, allowing fast, automated multiparametric analysis including measurements of spheroid diameter, perimeter and area. This application allows the generation of image segmentation around the spheroid, as shown in Figure 1b. Images are acquired at intervals and growth curves are rapidly and easily generated, showing linear volume increases up to 14 days following cell seeding. An example using U-87 MG glioblastoma is shown in Figure 1c. Small standard deviations demonstrate the reproducibility and robustness of the method.

We tested 40 tumor cell lines in total (Table 1) and classified spheroids according to their morphology, extending the original Ivascu classification [23] as follows: tight spheroids (for example, SF188 glioblastoma): tight, spherical and regular; compact aggregates (for example, MDA-MB-231 breast carcinoma): acceptable for three-dimensional studies but less regular; loose aggregates (for example, IGROV-1 ovarian carcinoma): irregular, friable aggregates (Table 1 and Additional file 1). The three-dimensional structure of compact and loose spheroids can be improved by the addition of Matrigel™ (BD Biosciences, Oxford, England) at the time of initiation, extending the range of tumor cells suitable for further study (Additional file 1).

**Table 1:** Tumor cell lines

Cell line	Tumor type	Three-dimensional morphology	Supplier	Medium
U-87 MG	GBM (adult)	T	ATCC	1
A172	GBM (adult)	T	ATCC	1
SF126	GBM (adult)	T	JCRB	1
LN-229	GBM (adult)	T	ATCC	1
SF268	GBM (adult)	C	NCI repository	1
SF188	GBM (pediatric)	T	Bax, 2009 [56]	2
KNS42	GBM (pediatric)	T	Bax, 2009 [56]	2
RES186	Astrocytoma (pediatric)	T	Bax, 2009 [56]	2
RES259	Astrocytoma (pediatric)	T	Bax, 2009 [56]	2
UW479	Astrocytoma (pediatric)	L	Bax, 2009 [56]	2
WT-CLS1	Wilm's kidney	T	CRUK Cell Line Services	1
WiT49	Wilm's kidney	T	Dr H Yeger, Hospital for Sick Children, Toronto	1
IMR-32	Neuroblastoma	T	ATCC	1
SH-SY5Y	Neuroblastoma	C	Dr L Chesler, ICR	3
KELLY	Neuroblastoma	C	Dr L Chesler, ICR	3
SHEP	Neuroblastoma	T	Dr L Chesler, ICR	3
U-2 0S	Osteosarcoma	C	ATCC	1
Detroit 562	SCC (pharynx)	T	ATCC	1
CAL 27	SCC (tongue)	T	ATCC	1
PECA/CA-PJ34	SCC (basaloid)	T	ECACC	4
PE/CA-PJ41^a^	SCC (oral)	C	ECACC	4
SIHN-011A	SCC (tongue)	C	O-charoenrat, 2001 [57]	1
LICR-LON-HN4	SCC (larynx)	T	Easty, 1981 [58]	1
SK-Mel-5	Melanoma	L	ATCC	1
NCI-H23	Lung	C	ATCC	3
MDA-MB-231^a^	Breast	C	ATCC	1
MCF-7	Breast	C	ATCC	1
DLD-1	Colon	T	ATCC	1
HCT 116	Colon	T	ATCC	1
RKO^a^	Colon	L	ATCC	1
HT-29	Colon	T	ATCC	1
SW620^a^	Colon	L	ATCC	1
PC-3^a^	Prostate	L	ATCC	3
A2780	Ovarian	L	ECACC	3
IGROV-1^a^	Ovarian	L	CRUK Cell Line Services	3
SK-OV-3^a^	Ovarian	L	ATCC	3
PANC-1^a^	Pancreatic	C	ATCC	1
MIA PaCa-2	Pancreatic	L	ATCC	1
Hep G2	Hepatocellular	C	ATCC	5
Hep 3B	Hepatocellular	C	ATCC	5

^a^Cell lines in which three-dimensional structure is improved by addition of Matrigel at spheroid initiation.

Medium: 1 = DMEM; 2 = DMEM:F12; 3 = RPMI; 4 = Iscove's Modified Dulbecco's Medium; 5 = Eagle's Minimum Essential Medium.

ATCC = American Type Culture Collection; C = compact aggregate; ECACC = European Collection of Cell Cultures; GBM = glioblastoma; JCRB = Japanese Collection of Research Bioresources; L = loose aggregate; SCC = squamous cell carcinoma; T = tight spheroid

To demonstrate the reproducibility in spheroid size, the coefficient of variation (CV) of spheroid volumes on day 4 was determined for selected highly malignant tumor cell lines that form tight spheroids. The intraplate CV was 5.53% to 7.15% for U-87 MG (adult glioblastoma), 4.97% to 10.57% for KNS42 (pediatric glioblastoma) and 7.59% to 10.59% for LICR-LON-HN4 (laryngeal squamous cell carcinoma). The interplate CV gave values of 17.88% for U-87 MG, 11.21% for KNS42 and 10.75% for LICR-LON-HN4 (n = 3, tested over three different batches of plates). Furthermore the spheroid volume distribution follows a Gaussian distribution (U-87 MG, Figure 1d). Similar results were obtained for other cell lines such as KNS42 and LICR-LON-HN4 (data not shown).

**Figure 2:**
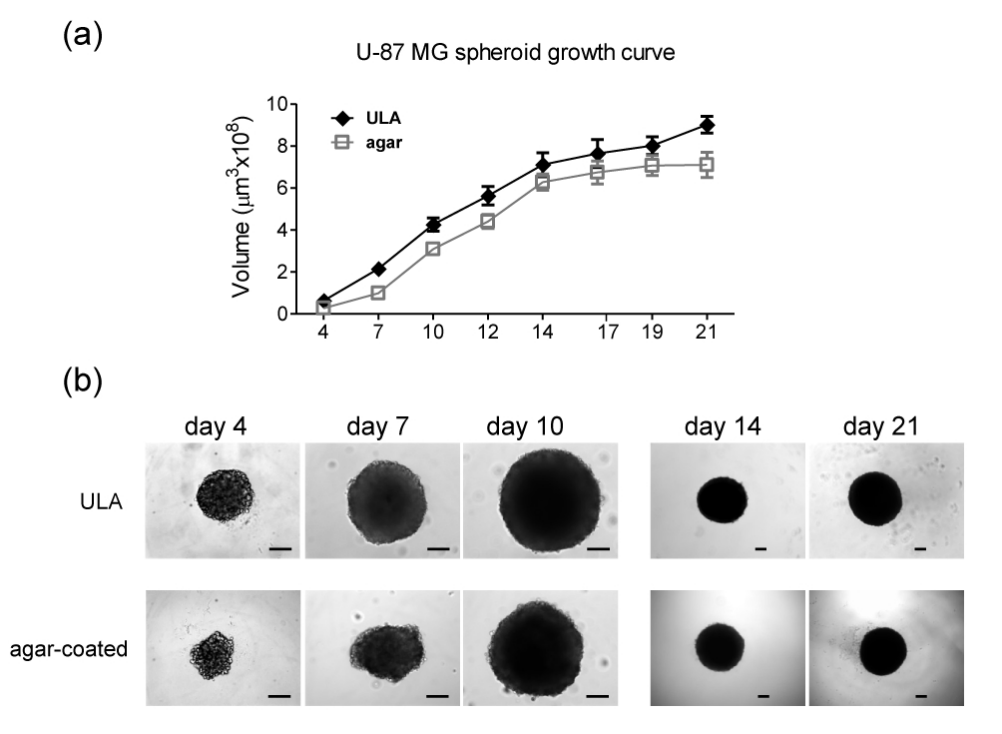
**Tumor spheroid growth: ultra-low attachment (ULA) 96-well round-bottomed plates vs agar-coated 96-well flat-bottomed plates**. **(a) **ULA 96-well round-bottomed plates and agar-coated 96-well flat-bottomed plates were used to generate U-87 MG spheroids (a single spheroid per well). Starting from day 4 post initiation, images were obtained at intervals using an inverted microscope. Analysis was carried out using Image-Pro Analyzer software and growth curves were obtained. Values are means ± SD (n = 16 spheroids/timepoint; a representative result of two independent experiments is shown). **(b) **Representative images of U-87 MG spheroids grown on ULA plates or on agar-coated plates at the indicated timepoints. Scale bar: 100 μm.

In order to compare our system to a standard conventional method, U-87 MG spheroids were initiated and cultured in parallel on agar-coated 96-well flat-bottomed plates [26] and in ULA 96-well round-bottomed plates (Figure 2). The agar-coated plates, unlike the ULA plates, proved unsuitable for automated image analysis on the Celigo cytometer (probably due to variations in the thickness of agar in each well). Thus, images were obtained at intervals using an Olympus IX 70 (Olympus Microscopy, Southend-on-SeaEssex, UK) inverted microscope. Analysis was carried out using Image-Pro Plus Analyzer software (Media Cybernetics, Inc., Bethesda, MD, USA; http://www.mediacy.com/index.aspx?page=IPP). Reproducibly sized spheroids (a single spheroid/well) were obtained in each system, with ULA spheroids growing to slightly larger sizes than the agar-based spheroids but both growth curves reached a plateau at around day 14 (Figure 2a).

**Figure 3:**
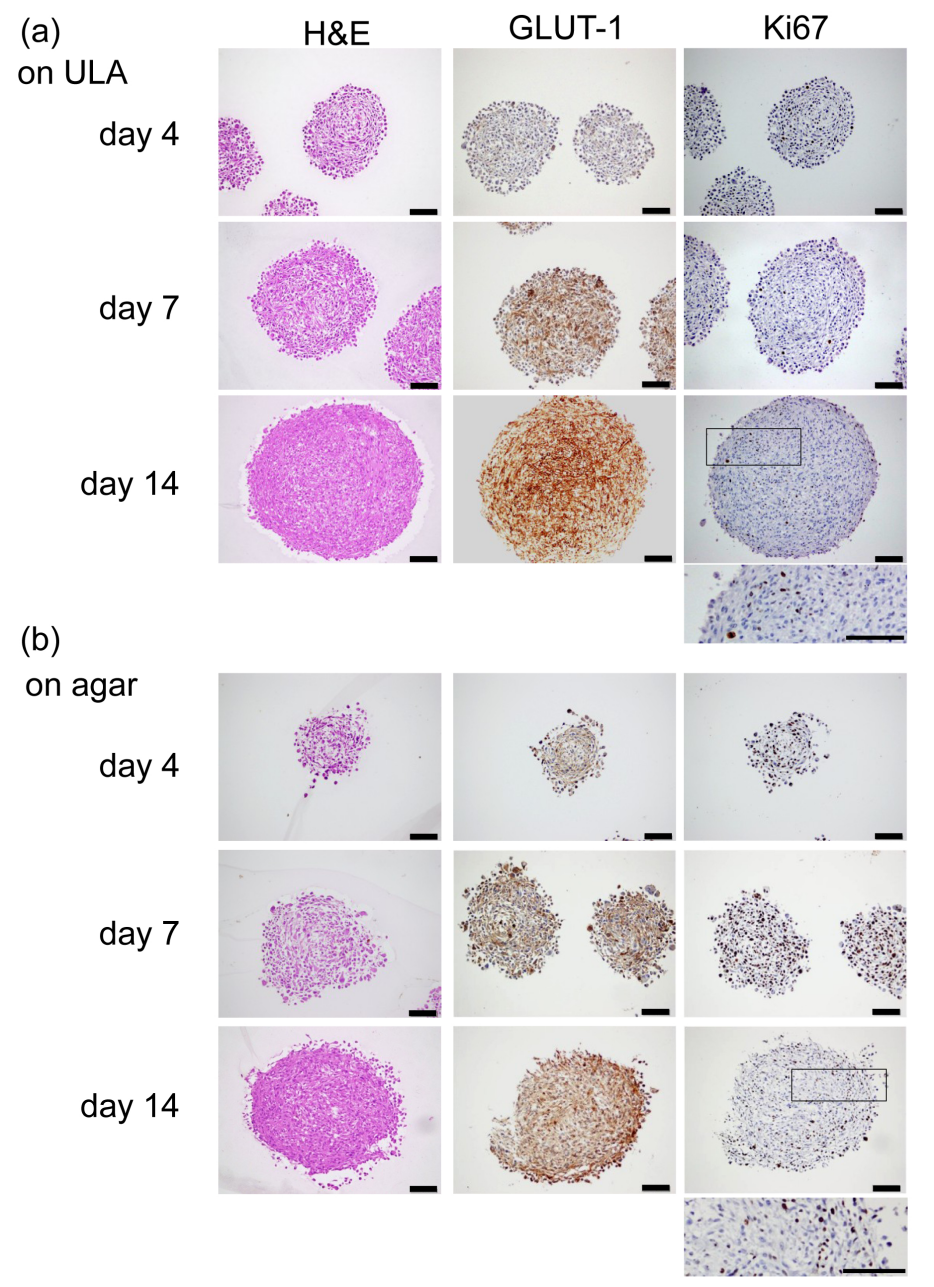
**Comparison of U-87 MG spheroids grown on ultra-low attachment (ULA) plates or agar: immunostaining**. Hematoxylin and eosin (H&E), glucose transporter 1 (GLUT-1) or Ki67 antibody staining of sections of U-87 MG spheroids grown for 4, 7 and 14 days on **(a) **ULA 96-well round-bottomed plates or **(b) **agar-coated 96-well flat-bottomed plates. Spheroids on (a) ULA plates show a tighter structure than spheroids grown on (b) agar, across all timepoints. H&E staining is homogeneous, while there was a gradient of proliferation (Ki67) in larger spheroids (day 14). A more intense staining of GLUT-1 was observed concomitantly with the age of the spheroids, irrespective of the means of generation (a, b). Scale bar 100: μm.

Spheroids grown on ULA plates showed a more compact structure than those on agar-coated plates (Figures 2b, 3a, b and Additional file 2a). In both cases, (suspension and agar plated) larger U-87 MG spheroids develop a gradient of proliferation as measured by Ki-67 staining [10] and a more intense glucose transporter 1 (GLUT-1) staining (Figure 3a, b) that correlates with tumor hypoxia [38]. Central necrosis was not evident in U-87 MG spheroids over this observation period although was seen in HCT 116 spheroids (data not shown). Thus, overall our system compares closely with conventionally-generated spheroids, but with the advantage that automated analysis and retrieval of spheroids for molecular analysis is more easily achieved with suspension cultures in ULA plates. U-87 MG spheroids were also grown on poly-Hema-coated 24-well plates and in a Rotary Cell Culture System (RCCS; Synthecon, El Rio Houston, TX, USA; http://www.synthecon.com/) (Additional file 2c) but multiple non-reproducibly sized spheroids were formed and hence these methods were not pursued further.

#### Three-dimensional tumor spheroid functional assays

**Figure 4:**
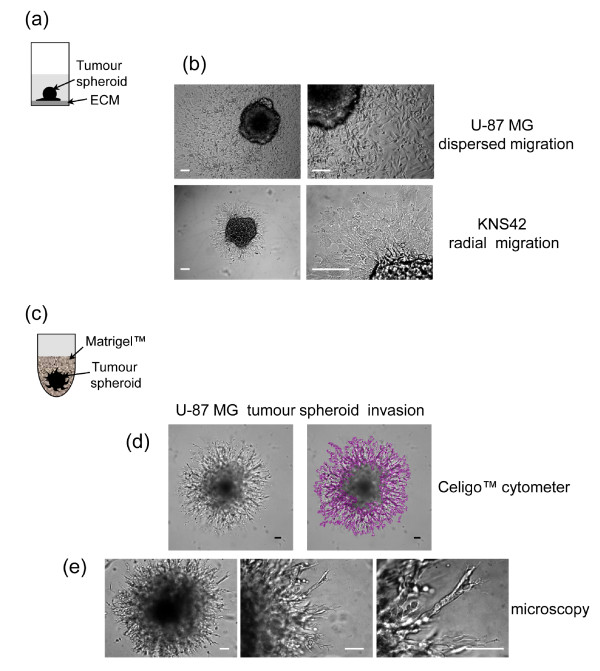
**Three-dimensional tumor spheroid-based functional assays: migration and invasion**. **(a) **Tumor spheroid-based migration is illustrated in a cartoon: a single day 4 tumor spheroid was transferred to each well of extracellular matrix (ECM)-coated 96-well flat-bottomed plate using a multichannel pipette. **(b) **Representative brightfield images of U-87 MG and KNS42 cell migration (48 h) are shown. **(c) **Tumor spheroid invasion into Matrigel is illustrated in a cartoon: a single tumor spheroid per well was embedded in Matrigel. **(d) **Representative images of a typical U-87 MG 'starburst' invasion pattern obtained with a Celigo cytometer. The Cell Counting Confluence application (1/16 field of view/well) produces a segmentation analysis that identifies the area occupied by invading cells. **(e) **Representative images obtained using a conventional inverted microscope show the U-87 MG invasion at low magnification and details of invadopodia advancing into the matrix shown at higher magnification. Scale bar: 100 μm.

**Figure 5:**
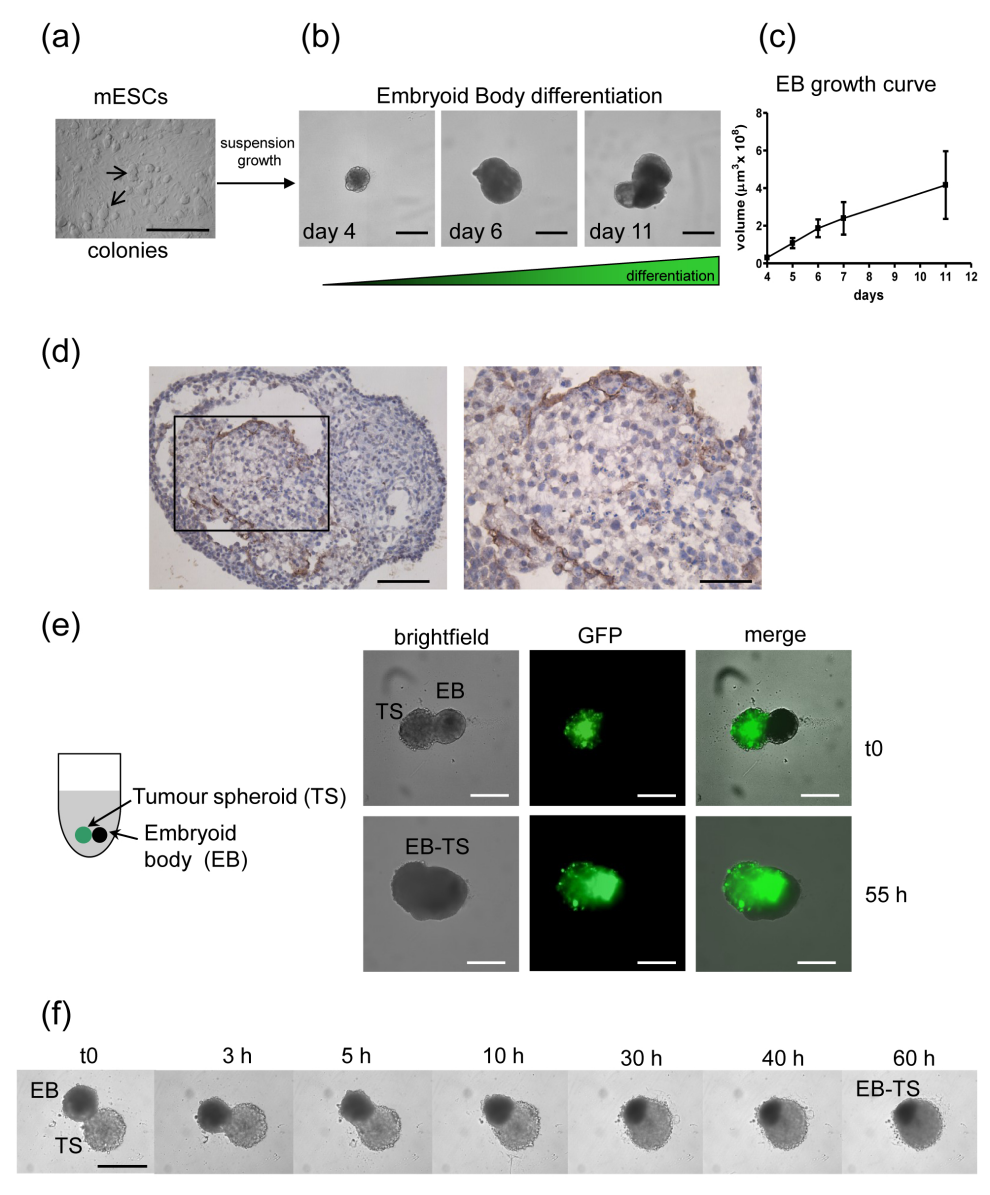
**Mouse embryonic stem cell (mESC) differentiation into embryoid bodies (EBs) and confrontation culture with tumor spheroids**. **(a) **R1 mES cells grown as colonies on feeder layers of mouse embryonic fibroblasts (MEFs) in the presence of leukemia inhibitory factor (LIF). **(b) **Suspension growth of mESCs on ultra-low attachment (ULA) 96-well round-bottomed plates allows spontaneous differentiation into EBs. Representative images of EBs are shown. Scale bar: 500 μm. **(c) **EB growth curve, determined by automated imaging analysis on a Celigo cytometer, (n = 12 EBs/timepoint). Values are mean ± SD. **(d) **CD34 staining of day 12 EBs shows endothelial differentiation (brown staining). Scale bar: 100 μm (left) or 50 μm (right). **(e) **Confrontation culture of green fluorescent protein (GFP)-transduced U-87 MG tumor spheroid (TS) and EB is represented in a cartoon on the left: a single TS and EB are cocultured in each well of a ULA 96-well round-bottomed plate. Representative images are shown at t0 and 55 h. Images were obtained on a Celigo cytometer. Scale bar: 500 μm. **(f) **Selected frames of a timelapse study show mutual invasion and coalescence of the two organoids. Images were obtained on an inverted microscope. Scale bar: 500 μm.

Another major advantage of our approach is its convenient extension to additional three-dimensional functional assays to address important cellular processes in tumor progression: (i) migration on extracellular matrix proteins (ECM), (ii) invasion into Matrigel and (iii) simultaneous tissue invasion/angiogenesis (by coculture with EBs) (Figures 4 and 5).

#### Migration on matrix protein

We utilized a tumor spheroid-based migration assay that resembles tumor cell dissemination from a solid microtumor or micrometastasis in terms of engagement of host stromal matrix proteins with characteristics that differ from isolated cells [39-41]. The same type of assay has been used previously in a lower throughput format and with more time-consuming methods for generation of tumor spheroids [42-44]. We successfully modified the assay for HT format by rapidly transferring tumor spheroids generated in ULA round-bottomed plates into gelatin-coated flat-bottomed 96-well plates (a single spheroid/well) using a multichannel pipette (Figure 4a). Within a few hours, tumor cells disseminate from the spheroid over the coated surface [44] and migration is recorded at intervals up to 72 h. Images can be obtained using an inverted microscope and analysis performed using Image-Pro Analyzer software by manually recording the leading edge of the migrating cells, which enables the software to calculate the area covered. This assay also provides valuable qualitative information on different cell migration patterns (Figure 4b). For example, U-87 MG cells show rapid, single cell dispersal resembling typical amoeboid migration [45,46]. In contrast, KNS42 cells move slowly and collectively in a radial pattern, reflecting a more mesenchymal phenotype [45,46] (Additional files 3 and 4).

#### Three-dimensional invasion into Matrigel

To measure three-dimensional tumor invasion (Figure 4c), spheroids are initiated as described above and on day 4 Matrigel is added to the wells. This provides a semisolid gel-like matrix into which cells extend invadopodia [47,48]; these processes contribute to cell movement and also matrix degradation via localized proteolysis [47]. Within a few hours, the invadopodia are apparent and invasion is monitored at intervals over 72 h. Image analysis is performed on a Celigo cytometer by using the Cell Counting Confluence application, which determines the area occupied by individual cells or cell clusters, identified by image segmentation around the invading cells (Figure 4d). The central location of each spheroid allows rapid and automated image analysis and by selecting 1/16 field of view per well the scan is completed within 10 minutes/plate. Alternatively, images can be obtained using an inverted microscope and analysis is performed as described for the migration. Microscopy additionally provides detailed images of the invading cell protrusions as shown in Figure 4e.

#### Tissue invasion and angiogenesis

The angiogenic switch is a critical step in tumor progression [49]. We modified a confrontation culture assay of spheroids and EBs [21] to mimic simultaneous tissue invasion and tumor angiogenesis *in vitro*. With their complex cellular heterogeneity, including differentiated endothelial cells, EBs represent a true tissue environment. The method is a simple three-step procedure: (1) culture of mouse embryonic stem cells (mESCs) (Figure 5a); (2) differentiation of mESCs into EBs (Figure 5b, c); (3) coculture of spheroids and EBs (Figure 5d, e). To optimize the assay for future HT target validation and/or drug evaluation studies, EBs were grown in ULA 96-well round-bottomed plates rather than Petri dishes [50] or spinner flasks [21], increasing EB reproducibility and significantly reducing the time needed for assay set-up. Fully automated cytometer image analysis shows linear growth curves with increased variability only during later stages of maturation (Figure 5b). Consistent with previous reports [21,50], we demonstrated endothelial differentiation by CD34 staining (Figure 5d).

Our confrontation culture is a significant enhancement of previous assays, since day 4 tumor spheroids are transferred directly into wells containing day 5 EBs (when endothelial cell differentiation begins [21]) and the system allows real-time analysis of subsequent interactions. Representative images from the Celigo cytometer show two distinct bodies at confrontation, with the U-87 MG spheroid identified by green fluorescent protein (GFP) fluorescence. The mutual integration process is complete within 50 to 60 h, as shown in the merged brightfield and fluorescent images (Figure 5e). Confrontation cultures can also be imaged by microscopy, allowing timelapse studies (Figure 5f and Additional file 5). Treatment with 17-AAG was shown to inhibit U-87 MG EB tissue invasion (Additional file 6). Tumor-stimulated angiogenesis is illustrated by immunohistochemical staining of EB-derived endothelial cells within tumor spheroids (Additional file 7).

Together, these assays provide a suite of novel, complementary functional assays with immediate relevance to cancer progression and enabling rapid evaluation of new therapeutic agents, as exemplified below.

### Exemplification of the methods for evaluation of anticancer agents

#### Evaluation of effects of signaling inhibitors on cell viability in three-dimensional vs two-dimensional cultures

In order to evaluate responsiveness in our suite of tumor spheroid assays, we first compared the sensitivity of cells in three-dimensional vs two-dimensional cultures to selected compounds using the CellTiter Glo cell viability assay which is quick, easy and reproducible. This was validated in pilot studies that determined optimum incubation time of the reagents, and showed good correlation between spheroid size and luminescent emission as well as spheroid size and the number of viable cells per spheroid (Additional file 8).

MDA-MB-231, U-87 MG, KNS42 and LICR-LON-HN4 cells were treated with 17-AAG (HSP90 chaperone inhibitor), PI-103 (PI3 kinase/mTOR inhibitor) or CCT130234 (PLCγ inhibitor) in two-dimensional and three-dimensional cultures, and concentrations inhibiting cell viability by 50% (GI_50_) were determined after 72 h. Tumor cells were generally less sensitive to compounds in three-dimensional than in two-dimensional cultures (Table 2 and Additional file 9). MDA-MB-231 P (parental variant) cells treated with 17-AAG showed a clear shift of the cell survival curve, resulting in a higher GI_50 _in three dimensions (Additional file 10a). This could reflect reduced compound access or pathophysiological differences in the response of hypoxic or more slowly cycling cells under these conditions (as in solid tumors *in vivo *[51]). This was not the case with CCT130234 in three dimensions (Additional file 10b). Furthermore, both U-87 MG and KNS42 were more sensitive in three dimensions to PI-103 (Table 2 and Additional file 9) confirming previous reports of increased sensitivity to PI3 kinase inhibitors in three dimensions [34]. This highlights the importance of accurately evaluating drug responses under appropriate conditions *in vitro *in order to avoid overestimating or underestimating the effect of compounds prior to *in vivo *studies.

**Table 2:** Concentration inhibiting cell viability by 50% (GI_50_) values for selected tumor cell lines treated with 17-*N*-allylamino-17-demethoxygeldanamycin (17-AAG), PI-103 or CCT130234

Inhibitor (μM)	Cell line	Two-dimensional	Three-dimensional
17-AAG:	U-87 MG	0.43 ± 0.13	0.82 ± 0.16
	KNS42	18.02 ± 6.42	81.43 ± 1.60
	MDA-MB-231 P	2.47 ± 0.83	2.33 ± 0.33
	MDA-MB-231 M	0.75 ± 0.10	6.19 ± 0.50
	LICR-LON-HN4	0.07 ± 0.06	0.38 ± 0.11
PI-103:	U-87 MG	0.49 ± 0.13	0.16 ± 0.03
	KNS42	2.15 ± 1.20	0.72 ± 0.34
	MDA-MB-231 P	2.39 ± 0.69	> 100
CCT130234:	U-87 MG	9.10 ± 1.13	16.40 ± 0.14
	MDA-MB-231 P	11.5 ± 3.32	8.50 ± 0.62

Cells cultured in two dimensions and three dimensions were treated with compounds for 72 h. A CellTiter Glo^® ^viability assay was performed and GI_50 _values were obtained. Values are means ± SD of the studies reported in Additional file 10 (apart from MDA-MB-231 P that when treated with PI-103 over the concentration range tested, did not achieve a GI_50_).

#### Tumor spheroid growth inhibition with 17-AAG and PI-103

**Figure 6:**
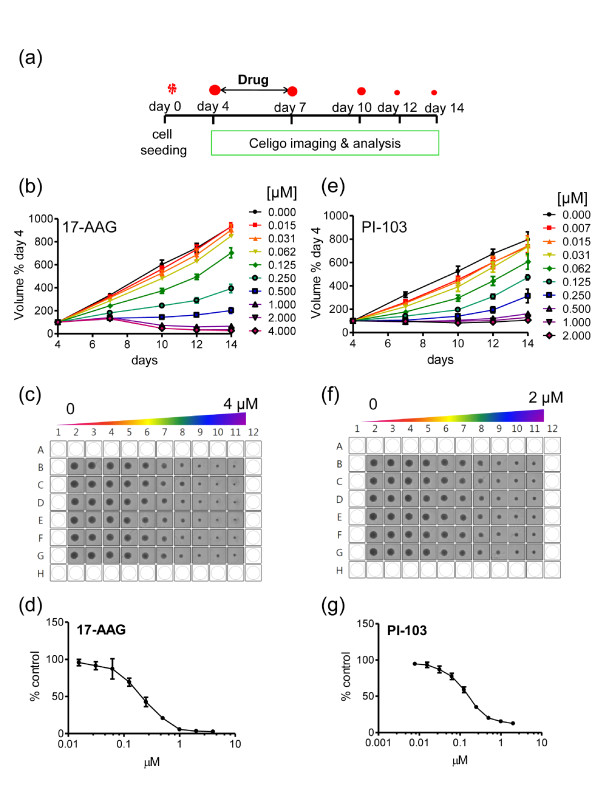
**17-N-Allylamino-17-demethoxygeldanamycin (17-AAG) and PI-103 concentration-dependent inhibition of tumor spheroid growth**. **(a) **Schematic illustration of tumor spheroid growth kinetics and compound treatment procedures. Spheroids were initiated on day 0, treated with compound or vehicle on day 4, and 50% medium replenishment performed on days 7, 10 and 12. A Celigo cytometer was used for automated imaging and analysis. **(b-g) **Day 4 U-87 MG spheroids were treated with 17-AAG (heat shock protein 90 (HSP90) inhibitor) or PI-103 (phosphatidylinositol-3-kinase (PI3K)- mammalian target of rapamycin (mTOR) inhibitor) with 1:2 serial dilutions (final concentrations 0 to 4 μM for 17-AAG and 0 to 2 μM for PI-103). Control spheroids were treated with vehicle. Values are means ± SD (n = 6) and a representative of three separate experiments for each agent is shown. Student t test with Welch's correction was performed relative to the 14-day values: (b) vehicle controls vs 0.015 and 0.031 μM not significant; vs 0.062 μM *P *< 0.01; vs all higher concentrations *P *< 0.001; (e) vehicle controls vs 0.007 to 0.031 μM not significant; vs all higher concentrations *P *< 0.001. Screen prints of plates for (c) 17-AAG or (f) PI-103, corresponding to day 14 of the above graphs are also shown. Concentration response curves at day 14 for 17-AAG (d) and PI-103 (g) are shown. Values are means ± SD (n = 6).

For evaluation of the effects of molecularly targeted agents on three-dimensional tumor growth kinetics, 4-day-old spheroids (for example, U-87 MG) were treated with compounds for 72 h. Medium replenishment and imaging was performed as shown on the schematic protocol in Figure 6a. 17-AAG and PI-103 induced concentration-dependent growth inhibition (Figure 6b, d for 17-AAG and Figure 6e, g for PI-103).

This assay allows dynamic effects of compounds to be measured over time; a significant advantage compared to standard endpoint assays in two dimensions. Moreover, it can demonstrate whether a single administration of the agent is sufficient to inhibit spheroid growth or induce 'regression' as shown in Figure 6b. Note the excellent reproducibility of the spheroids on each plate across the six replicates/conditions (Figure 6c, f) and the Gaussian distribution of the spheroid volume that is maintained over the observation period, not only in controls but also in treated U-87 MG spheroids (Additional file 11). Tumor spheroid growth in this system is thus characterized by small standard deviations, which facilitate quantitative analysis and the ability to sensitively discern statistically significant differences between test and control conditions. Similar results of dose response inhibition on tumor spheroid growth were obtained with further cell lines, for example, KNS42 and LICR-LON-HN4, treated with PI-103 and 17-AAG, respectively (Additional file 12).

KNS42, LICR-LON-HN4 and U-87 MG generate tight spheroids, ideal for fully automated growth kinetic assays. With spheroids of looser morphology, or where agents cause dissociation of the three-dimensional structure, a Celigo cytometer still provides fully automated imaging but does not always provide accurate size determination. Images can then be imported into Image-Pro Analyzer software and manually manipulated for data analysis (data not shown). Alternatively, if no cytometer is available, images can be obtained using an inverted microscope and analyzed on Image-Pro Analyzer software. The results obtained using both methods are highly comparable; thus the latter is a perfectly acceptable (lower throughput) alternative. Two comparative examples are shown in Additional file 13 for U-87 MG and LICR-LON-HN4 spheroids treated with 17-AAG. Concentration-dependent inhibition trends are fully reproducible between the two imaging/analysis systems as demonstrated by a head-to-head comparison using LICR-LON-HN4 (Additional files 12 and 13).

#### HSP90 and PLCγ inhibitors prevent tumor dissemination on matrix protein

**Figure 7:**
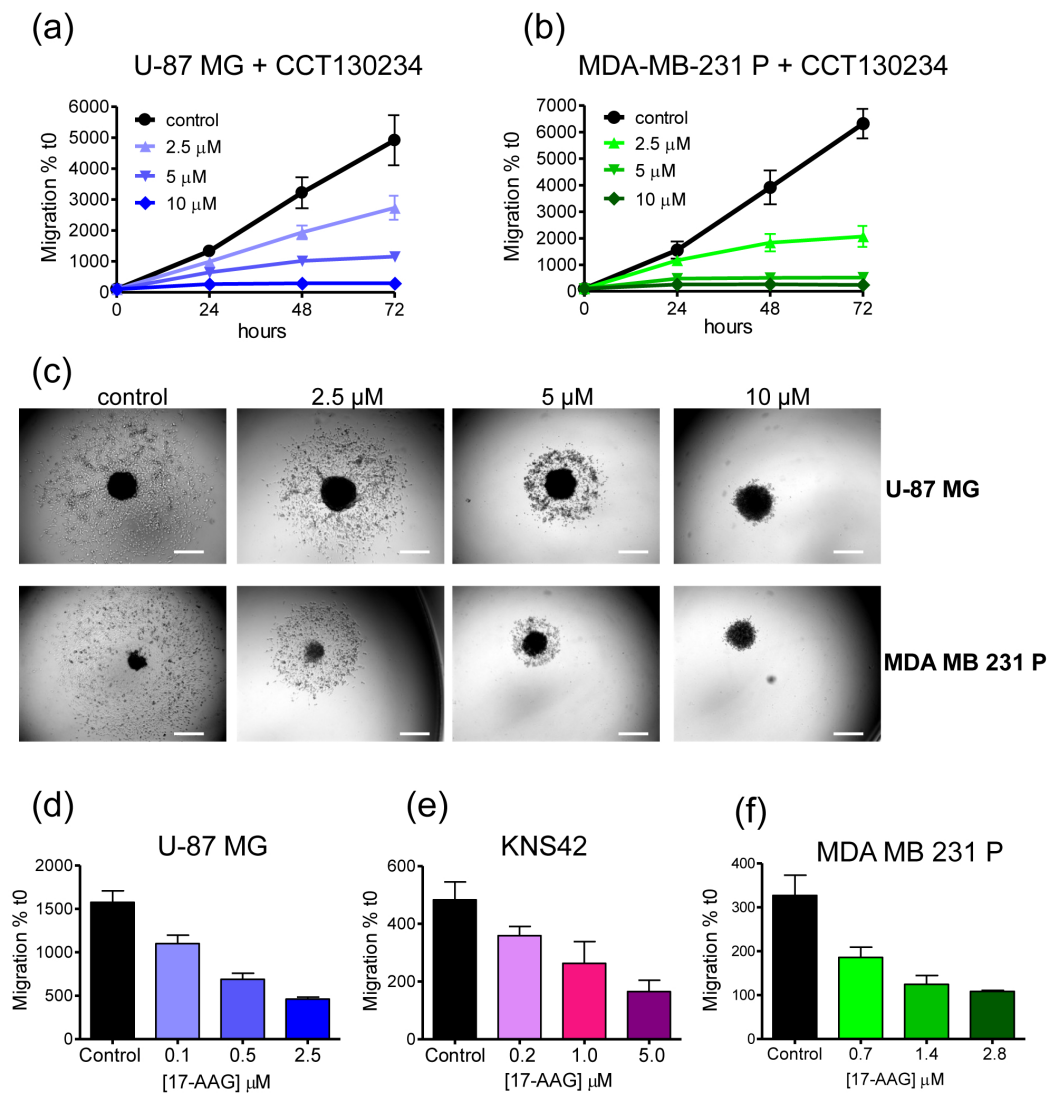
**CCT130234 and 17-*N*-allylamino-17-demethoxygeldanamycin (17-AAG) concentration-dependent inhibition of tumor dissemination on matrix protein**. **(a-c) **Day 4 U-87 MG and MDA-MB-231 P (parental variant) spheroids were placed on gelatin-coated plates and treated with the phospholipase C (PLC)γ inhibitor (CCT130234) over a range of concentrations. Controls were treated with vehicle. Images were captured at intervals (from t0 to 72 h) using an inverted microscope and analyzed with Image-Pro Analyzer software. Representative results of three separate experiments are shown for (a) U-87 MG and (b) MDA-MB-231 P. Values are means ± SD; (n = 12). Student t test with Welch's correction was performed relative to 72 h values: (a) vehicle control vs 2.5 to 10 μM *P *< 0.001; (b) vehicle control vs 2.5 to 10 μM *P *< 0.001. (c) Representative images for both cell lines at 72 h are shown. Scale bar: 500 μm. **(d) **U-87 MG, **(e) **KNS42 and **(f) **MDA-MB-231 P spheroids were treated with 17-AAG over a range of concentrations and the area of migration at 24 h post treatment quantified. Values are means ± SD; n = 6. Student t test with Welch's correction was performed: (d) vehicle control vs 0.1 to 2.5 μM *P *< 0.001; (e) vehicle control vs 0.2 μM **P < 0.01, vs 1 and 5 μM *P *< 0.001; (f) vehicle control vs 0.7 to 2.8 μM *P *< 0.001. A representative example of three separate experiments is shown for each cell line.

The tumor spheroid-based migration assay (Figure 4a) was adopted to mimic cell motility mediated by cell-substrate interactions. We previously reported that PLCγ1 is important in integrin-mediated cell migration [37]. To exemplify the power of the assay, we show here that an in-house PLCγ inhibitor (CCT130234) reduces the migration of both U-87 MG and MDA-MB-231 tumor cells at sub-GI_50 _concentrations (Figure 7a-c and Table 2).

HSP90 is also involved in tumor cell migration through client proteins including c-MET, SRC and FAK [35]. The HSP90 inhibitor 17-AAG potently inhibited migration of U-87 MG, KNS42 and MDA-MB-231 cells in a concentration-dependent manner as early as 24 h (Figure 7d-f and Table 2). The fact that inhibitory effects are manifest at low concentrations and short time intervals is of particular importance, indicating selective effects on tumor cell migration at least partly independent of proliferation.

#### 17-AAG inhibits three-dimensional tumor spheroid invasion into Matrigel

**Figure 8:**
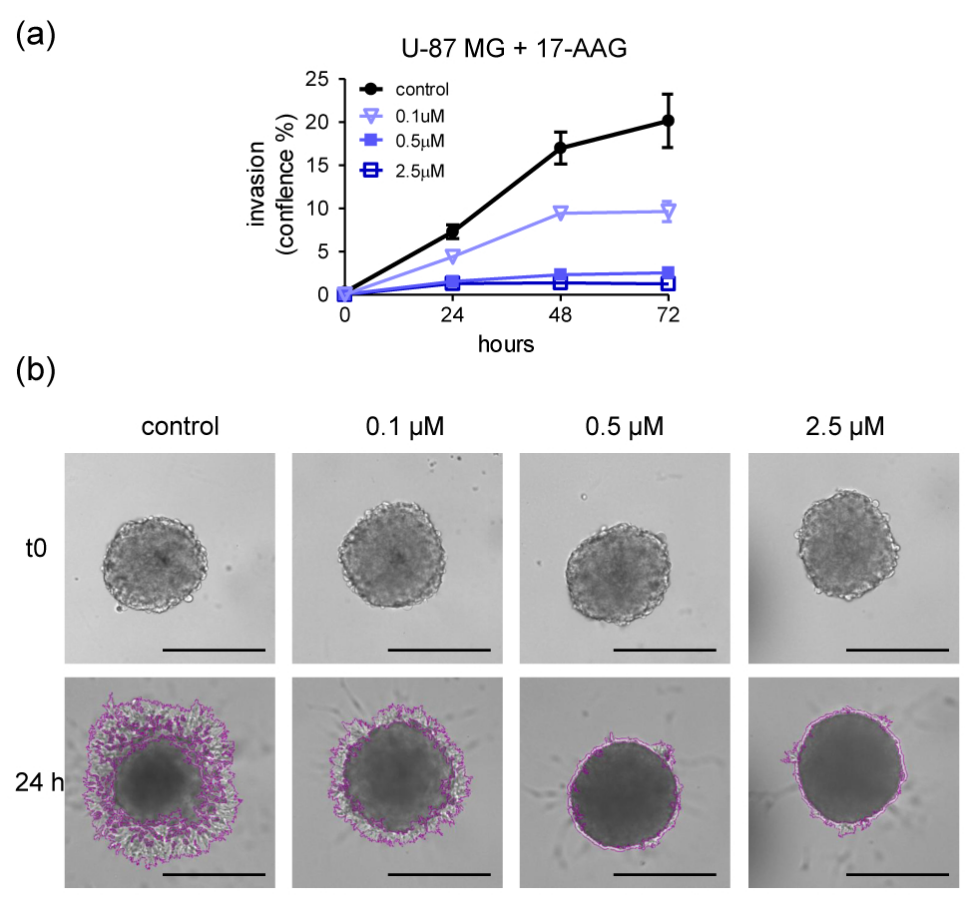
**17-N-Allylamino-17-demethoxygeldanamycin (17-AAG) mediated concentration-dependent inhibition of tumor spheroid invasion into Matrigel**. **(a, b) **Day 4 U-87 MG spheroids were embedded into Matrigel for three-dimensional invasion. 17-AAG was used over a range of concentrations and control spheroids were treated with vehicle. Automated image analysis was carried out at intervals from t0 to 72 h on a Celigo cytometer using the Colony Counting Confluence application scanning 1/16 field of view/well. Invasion was normalized to t0. (a) A representative example of three separate experiments is shown. Values are means ± SD; n = 8. Student t test with Welch's correction was performed relative to 72 h values: vehicle control vs 0.1 to 2.5 μM *P *< 0.001. (b) Representative images of U-87 MG spheroid invasion obtained on a Celigo cytometer show the reproducibly sized spheroids at t0 and the typical segmentation around the invading cells with a clear inhibition by the 17-AAG at 24 h (also seen at concentrations below the concentration inhibiting cell viability by 50% (GI_50_) for this cell line (see Table 2)). Images of one field of view/well were cropped and magnified (20 ×) to allow visualization of the invasion. Scale bar: 500 μm.

Another aspect of tumor progression is the ability of cells to invade basement membranes and connective tissue, enabling escape from the primary tumor and, in some cases, subsequent metastasis. U-87 MG glioma spheroids (representing a locally invasive tumor type) were embedded in Matrigel and treated with 17-AAG. Tumor cell invasion was monitored up to 72 h, although effects of the compound were apparent after just 24 h and invasion was inhibited at sub-GI_50 _concentrations (Figure 8a, b and Additional file 14a). A Celigo cytometer was used for a fully automated image analysis (Figure 8a) and representative images with the segmentation analysis around the invading cells are shown in Figure 8b at the 24 h timepoint. Additionally, similar results are obtained using the Celigo cytometer for automated imaging only, with the analysis performed on Image-Pro Analyzer software as an example of lower throughput (Additional file 14a). As indicated previously, a non-automated image capture and analysis method (microscopy and Image-Pro Analyzer software) can similarly be applied. This is illustrated by the inhibitory effects of 17-AAG on invasion of spheroids formed by highly malignant MDA-MB-231 M (metastatic variant) triple negative breast cancer cells (Additional file 14c).

## Discussion

Building on the pioneering work of Friedrich *et al. *[26] and others, here we demonstrate a novel method for the rapid generation and quantitative analysis of tumor spheroid growth in HT format, with additional applications in further important aspects of the malignant phenotype. The assays can readily be established in any laboratory without specialized equipment and negate the need to purchase preformed spheroids, which may only be available for a limited number of cell lines. Although the quantitation is significantly enhanced by the use of automated cytometry, we have also deliberately exemplified its utility using standard microscopy to broaden its accessibility to the research community.

Recent advances in the development of three-dimensional cultures for cancer drug evaluation have focused mainly on methods that avoid cell surface adhesion and promote cell-cell attachment [12,26,52]. Most are based on cellular aggregation on agarose-coated flat-bottomed plates [26,28], poly-Hema-coated round-bottomed or V-bottomed plates [52] or hanging drops [25]. Although able to generate reproducibly-sized spheroids, all such methods rely on time-consuming procedures or the need to transfer spheroids from delicate hanging drops to plates for further analysis. More recently, advances in microtechnologies have engineered plates for low binding of cells to the surface [30].

In pilot studies we compared different techniques for generating spheroids (bioreactor, agar-coated 96-well plates, poly-Hema-coated plates). We found that the ULA 96-well round-bottomed plate method is the least time consuming and generates the most reproducible spheroids. We have carried out a direct comparison with agar-coated plates and demonstrated that the tumor spheroids grown in ULA plates have equivalent growth rates, comparable gradients of proliferation and increased hypoxia in older spheroids but in general present a more compact and uniform structure. Also ULA plate-generated spheroids have the significant advantage that they are ideally suited to automated image analysis using a Celigo cytometer. Overall, the system (plus the additional simple extensions to key functional assays) represents an important advancement towards the routine use of three-dimensional cultures for preclinical oncology drug development.

HT three-dimensional growth assays must satisfy several key requirements: large scale, speed, simplicity, reproducibility and automation (both for set-up as well as readouts and data analysis). Here, we demonstrate that combining the use of ultra-low attachment round-bottomed plates with a Celigo cytometer, it is possible to utilize three-dimensional tumor spheroid assays in drug discovery projects in HT format. Building on this basic growth kinetic assay, we also successfully optimized *de novo *three-dimensional tumor spheroid-based functional assays in 96-well plates, for relatively HT formats: *in situ *three-dimensional tumor spheroid invasion into Matrigel, migration of cells from tumor spheroids on matrix proteins (also enabling detailed phenotypic analysis), and coculture of tumor spheroids with EBs representing tissue invasion with reciprocal angiogenesis.

In contrast to previously reported invasion assays [53,54], our method avoids the need to move spheroids and allows true three-dimensional invasion rather than superficial interactions between spheroids placed on top of (or between) layers of matrix proteins. In the complementary assays of cell migration and tissue invasion/angiogenesis, preformed tumor spheroids are easily and quickly transferred onto matrix-coated wells or into coculture with EBs (uniformly generated using the same ULA plates as for spheroids), in a single step using multichannel pipettes. Our methods significantly accelerate and simplify the assays, but at the same time generate highly reproducible results. Furthermore, they combine real-time quantitative kinetic analyses with options for more detailed morphological and molecular investigations: for example, qualitative determinations of modes of cell motility; recovery of spheroids for genetic/phenotypic or pharmacodynamic analyses, target validation using cells in which genes of interest are genetically repressed or mutated.

The assays can easily be modified further according to specific requirements: migration/haptotaxis on different matrix proteins such as collagen, fibronectin, laminin, on endothelial cell monolayers, or (for glioblastomas) on recently described nanofiber scaffolds [15]: invasion into alternative matrixes (for example, collagen) or, to avoid animal tissue derivates, fully synthetic biopolymers; microenvironment-enriched spheroids incorporating endothelial cells, immune cells and/or stromal cells [10]. The current spheroid-EB coculture is designed to mimic xenograft tumor transplant systems (human tumors encountering mouse tissue/vasculature), the mainstay of preclinical drug evaluation studies, but human ESCs could be substituted to generate a fully human three-dimensional confrontation culture system.

In addition to optimization and streamlining of spheroid generation and a suite of functional assays, we have successfully automated imaging and quantitation of real-time spheroid growth and also invasion using a Celigo cytometer. We are currently developing further protocols for fully automated imaging and analysis of the migration and the coculture assays. These, together with the tumor spheroid growth kinetic and viability assays will allow a complete analytical package to bridge the gap between simple two-dimensional proliferation assays and *in vivo *studies. We are currently using these assays in a number of target validation and cancer drug discovery projects where our data suggest that they have the ability to reflect *in vivo *activity. Once fully validated for a wider range of agents and shown to have predictive value for therapeutic efficacy, we believe such assays will become a mainstay of the preclinical functional tumor assay portfolio and reduce the need for extensive testing in animal tumor models.

## Conclusions

We provide a comprehensive suite of simple, reproducible three-dimensional tumor spheroid models that recapitulate *in vitro *some of the key hallmarks of cancer and that at the same time can provide a dynamic, automated, quantitative imaging and analysis compatible with high-throughput preclinical studies. We provide evidence that our methods have the potential to enhance target selection through rapid functional screening assays and the effective triaging of drug candidates prior to *in vivo *studies.

## Methods

### Cell lines and culture conditions

For the majority of our studies, four tumor cell lines representative of highly invasive cancers were selected: glioblastoma (U-87 MG, KNS42); oral squamous cell carcinoma (LICR-LON-HN4) and triple negative breast carcinoma (MDA-MB-231). Additional cell lines were included in spheroid formation trials to cover a wide range of tumor types.

Tumor cell lines (Table 1) were grown in tissue culture flasks under standard conditions (37°C, 5% CO_2_, 95% humidity). All media (Table 1) were as recommended by the supplier of the cells and were supplemented with 10% fetal calf serum (FCS), with the exception of the DLD-1 medium that was additionally supplemented with 1 × non-essential amino acids (NEAA). Media were routinely changed twice weekly. When subconfluent, cell monolayers were passaged using TrypLE Express Phenol Red, (Gibco, Life Technologies Ltd. Paysley, UK).

mES R1 cells were grown on mitomycin-C treated mouse embryonic fibroblast (MEF) feeder layers in ESC medium: Dulbecco's modified Eagle medium (DMEM) (4.5 g/l D-glucose; sodium pyruvate), supplemented with 15% FCS, 1 × penicillin, streptomycin and neomycin antibiotic mix (Gibco, Life Technologies Ltd. Paysley, UK), 100 μM β-mercaptoethanol (Sigma-Aldrich Company Ltd., Dorset, England), 1 × NEAA plus 1,000 U/ml leukemia inhibitor factor (LIF). Medium was changed 24 h after seeding (1.4 × 10^5 ^cells/ml, 5 ml/60 mm dish) and cells were passaged 2 to 3 days later. All media and NEAA were purchased from Gibco (Life Technologies Ltd. Paysley, UK), and FCS from Biosera.

### Generation and analysis of tumor spheroids

For spheroid generation, 200 μl/well of cell suspensions at optimized densities (0.5 × 10^4 ^cells/ml for U-87 MG, KNS42 and LICR-LON-HN4; 1.5 × 10^4 ^for MDA-MB-231 P and M variants) were dispensed into ULA 96-well round-bottomed plates (Corning B.V. Life Sciences, Amsterdam, The Netherlands) using a multichannel pipette. Plates were incubated for 4 days at 37°C, 5% CO_2_, 95% humidity. Where indicated, optimal three-dimensional structures were achieved by addition of 2.5% Matrigel as previously described [52]. Fully automated image analysis of tumor spheroids was carried out on a Celigo cytometer (Cyntellect Inc, San Diego, CA, USA; http://www.cyntellect.com/content/products/celigo/index.html), which is equipped with a 4-megapixel CCD camera with an F-theta scan lens (1 μm/pixel, 0.25 NA, 3.5 ×). Images were acquired and analyzed by using the Colony Counting Embryoid Body application with the option to scan 1/16 field of view/well. The width of 1 field of view (FOV) is 975 pixels/2,057 μm and image file size is 0.41 MB. Further technical details of imaging parameters are shown in Additional file 15.

For the lower throughput method, images were captured using an inverted microscope (Olympus IX 70, (Olympus Microscopy, Southend-on-SeaEssex, UK) equipped with a CCD camera (QImaging, Surrey, BC, Canada and imported into Image-Pro Plus Analyzer software (Media Cybernetics, Inc., Bethesda, MD, USA; http://www.mediacy.com/index.aspx?page=IPP) and by either using macros or manually, multiparametric analysis was performed. In both cases, the radius of each tumor spheroid was used to calculate the volume (μm^3^): V = 4/3 π r^3^.

For comparison with our system, U-87 MG cells were also used to generate spheroids using conventional methods as follows: (1) agar-coated 96-well flat-bottomed plates (BD Biosciences, Oxford, England) as previously described [26] (0.5 × 10^4 ^cells/ml as in the ULA 96-well round-bottomed plates); (2) poly-Hema-coated 24-well plates (2 × 10^5 ^cells/ml, 1 ml/well). A stock solution of 6 mg/ml poly-Hema (Sigma-Aldrich Company Ltd., Dorset, England) in 95% ethanol was prepared and diluted 1:10 in ethanol. A total of 100 μl/well was dispensed and left to dry before cell addition; (3) RCCS (10^5 ^cells/ml, 10 ml/disposable vessel).

In all cases, an inverted microscope was used for image analysis as described above.

### Tumor spheroid growth kinetics and treatment with test compounds

Spheroids were generated as described above. Growth kinetics and inhibition assays were performed as previously reported [26] but with significant modifications. Briefly, spheroid size was measured up to 14 days after initiation. A 50% medium replenishment was performed on days 4, 7, 10 and 12 using a multichannel pipette. Image analysis was performed on a Celigo cytometer or by microscopy as described. Where indicated, day 4 tumor spheroids were treated with 17-AAG (Invivogen, San Diego, CA, USA), or PI-103 (Charnwood Molecular Ltd., Loughborough, UK). Control spheroids were treated with appropriate vehicle. Then, 72 h following compound addition, 50% medium replenishment was performed as described above. Responses were evaluated by spheroid volume measurements at regular intervals.

### Tumor spheroid-based migration assay on matrix protein

Flat-bottomed, 96-well plates (Corning B.V. Life Sciences, Amsterdam, The Netherlands) were coated with 0.1% (v/v) gelatin (Sigma-Aldrich Company Ltd., Dorset, England) in sterile water for 1 h at 37°C. A total of 200 μl/well of culture medium supplemented with 2% (v/v) FCS was then added. For compound evaluation studies, medium contained 1.5 × the final concentration of 17-AAG or PLCγ inhibitor CCT130234, in a dilution series. Controls were treated with vehicle.

A total of 100 μl medium was removed from each well containing 4-day spheroids, and the remaining medium including the spheroids transferred into the prepared 'migration' plate using a multichannel pipette (final volume 300 μl). Spheroids were allowed to adhere and images were obtained at t0, 24, 48 and 72 h, using an inverted microscope as described. Effects of compounds were analyzed by measuring the area covered by migrating cells using Image-Pro Analyzer software to track the migration front. Data were normalized to the initial size of each spheroid at t0. For timelapse experiments, images were recorded every 15 minutes over a period of 70 h and videos edited using AVS Video Editor (Online Media Technologies Ltd., London, Uk).

### Tumor spheroid-based Matrigel invasion assay

A total of 100 μl medium was removed from wells containing 4-day spheroids and 100 μl Matrigel was gently added. When Matrigel solidified, 100 μl of culture medium was added on top. For compound evaluation studies, 17-AAG or vehicle was added to both the Matrigel and the overlying medium. U-87 MG spheroids were treated on the day of assay initiation while MDA-MB-231 spheroids were pretreated for 24 h and during the assay. From t0 and at intervals up to 72 h, automated image analysis was carried out for U-87 MG on a Celigo cytometer, using the Cell Counting Confluence application. Due to the central location of the spheroid in each well, only 1/16 of the field of view needed to be imaged. The width for 1 FOV is 1,958 pixels/2,055 μm and image file size is 1.55 MB. A segmentation analysis around the invading cells was produced and invasion measured as the area covered by the invading cells (percentage confluence) in the scanned field of view. Analysis settings must be adjusted for each cell type in order to achieve an optimal image segmentation that also reflects the degree of invasion. Alternatively, images were imported and analyzed on Image-Pro Analyzer software as described above. Finally, to demonstrate that equivalent data can be generated in the absence of a sophisticated cytometer, microscopic imaging and analysis on Image-Pro Analyzer software was performed using MDA-MB-231 M cells. Further technical specifications for both imaging methods are provided in Additional file 15.

### EB differentiation and tumor spheroid-EB confrontation culture

EB formation and cell differentiation was performed as described [50] with some modifications. R1 cells (1 to 5 × 10^3 ^cells/ml) in ESC medium containing 50 ng/ml vascular endothelial growth factor (VEGF) (Sigma-Aldrich Company Ltd., Dorset, England) were plated in ULA 96-well round-bottomed plates, 200 μl/well. A 50% medium refresh was performed daily from day 3 to day 14. Automated image analysis was performed using a Celigo cytometer as described and growth curves were generated.

For confrontation culture experiments [21], day 4 tumor spheroids and day 5 to day 7 EBs were used. Using a multichannel pipette, 100 μl of culture medium was removed from each well of the spheroid plate and replaced with 100 μl of ESC medium. This procedure was repeated three times, and after the last wash, remaining medium containing a single spheroid/well was transferred into the EB ULA plate to give a final volume of 200 μl/well, with one spheroid and one EB in each well. Confrontation cultures were incubated at 37°C in 5% CO_2 _for 60 h. To exemplify responses to a targeted agent, cocultures were treated with 17-AAG (5 μM) or with vehicle (control). For timelapse studies, videos were acquired on the Olympus microscope as described above. Images were recorded hourly over 65 h. Where indicated, GFP-transduced U-87 MG tumor spheroids were used and brightfield and fluorescence images of the confrontation cultures were obtained over 55 to 60 h on a Celigo cytometer, using the Expression Analysis application with the option to scan 1/16 field of view/well.

### Determination of coefficient of variation (CV) and frequency distribution of tumor spheroid size

During assay optimization, the reproducibility of spheroid size was measured for U-87 MG, KNS42 and LICR-LON-HN4 cells by determining their volumes on day 4. The intraplate and interplate CV for each cell line was calculated in at least three separate experiments over three different batches of plates. The frequency of spheroid volumes was plotted to illustrate Gaussian distribution of the sample for U-87 MG spheroids on day 4 (untreated) and on day 14 (controls and compound treated) in a representative growth kinetic study.

### Cell viability assay

Cell viability was measured using a CellTiter-Glo Luminescent Cell viability assay (Promega, Madison, WI, USA). In pilot studies to determine optimal incubation times, day 4 U-87 MG spheroids were incubated for 10, 30 and 60 minutes with CellTiter Glo reagents; all gave comparable results, hence a 10 minute incubation time was adopted for later studies. U-87 MG, KNS42, MDA-MB-231 and LICR-LON-HN4 spheroids were established as described. For cell monolayers (two-dimensional), cells were plated into 96-well black-sided flat-bottomed plates (Corning B.V. Life Sciences, Amsterdam, The Netherlands) at the following densities: 600 cells/well (U-87 MG, KNS42); 2,000 cells/well (MDA-MB-231) and 3,000 cells/well (LICR-LON-HN4). The outer wells of the plates were filled with phosphate-buffered saline (PBS) to reduce the effects of evaporation. Then, 4 days later, three-dimensional and two-dimensional cultures were treated with a range of concentrations of 17-AAG, PI-103, CCT130234 or appropriate vehicle. Cultures were incubated for 72 h and the CellTiter-Glo assay kit was used following the manufacturer's instructions. After 10 minutes of incubation with the CellTiter-Glo reagent, three-dimensional cultures were pipette mixed, aspirated with multichannel pipettes and transferred into black-sided, flat-bottomed plates (Corning B.V. Life Sciences, Amsterdam, The Netherlands) for luminescence measurement on a Synergy 2 SL Luminescence microplate reader (BioTek, Potton, UK) to generate GI_50 _values.

### Immunohistochemistry

Tumor spheroids (TSs), EBs and TS-EB cocultures were processed for immunohistochemistry as follows. Three-dimensional structures were collected in V-bottomed 15 ml Falcon tubes and allowed to sediment. Supernatant was removed by gentle aspiration and pellets washed once with PBS. After repeated sedimentation, supernatant was removed and 4% paraformaldehyde (PFA) was added and left overnight. The next day, PFA was removed and, using a warmed p1000 pipette tip, 1 ml of liquid agarose (4% w/v in sterile water) was gently added to the three-dimensional structures that were then collected and transferred into Tissue-Tek^®^Cryomolds^® ^(Sakura Finetek UK Ltd., Thatcham, UK). Solidified blocks were transferred into 50% ethanol for 1 h and then 80% ethanol before embedding in paraffin. Sections of 4 μm were cut using a semiautomated microtome HM 350 S (Microm International GmbH, Walldorf, Germany); the tissue sections were deparaffinized and rehydrated in water. Immunohistochemistry was performed as previously described [55] and sections were stained for Ki67 (monoclonal antibody, clone MIB1, Dako UK Ltd., Ely, UK), GLUT-1 (no. 07-1401, Millipore UK Ltd., London, UK) and CD34 (monoclonal antibody, clone MEC14.7, Abcam, Cambridge, UK). Slides were counterstained with hematoxylin and eosin and coverslipped with DPX mountant for microscopy (VWR Int., Lutterworth, UK).

### Statistical analyses

The Student t test with Welch's correction was performed where indicated using Prism5 (Graphpad Software, La Jolla, CA, USA; http://www.graphpad.com/welcome.htm). Values are expressed as means ± SD. *P *values ≤ 0.05 were considered statistically significant. For correlations between spheroid size (volume) and luminescent signal as well as for spheroid size and number of viable cells, Spearman correlation analysis was performed.

## Competing interests

The authors declare that they have no competing interests.

## Authors' contributions

MV, SG, FB, LP, CL and MZ performed the experiments and data analyses; WC established the optical imaging procedures; MM and DH optimized, performed and evaluated immunohistochemistry and provided statistical advice; MV and SE devised the studies and wrote the manuscript. All authors read and approved the final manuscript.

## Supplementary Material

Additional file 1 **Classification of three-dimensional tumor spheroid morphology**. Representative examples are shown: **(a) **SF188: tight spheroid; **(b) **MDA-MB-231 P: compact aggregate; **(c) **IGROV-1: loose aggregate; **(d) **IGROV-1 compact aggregate upon Matrigel addition. Scale bar: 200 μm. Click here for file

Additional file 2 **Generation of tumor spheroids: comparative methods**. U-87 MG spheroids were generated in **(a) **U-bottomed 96-well ultra-low attachment (ULA) plates, **(b) **flat-bottomed 96-well agar-coated plates, **(c) **24-well poly-Hema-coated plates and **(d) **in a Rotary Cell Culture System (RCCS; Cellon). In (a) and (b) one spheroid/well was obtained with a tighter structure in the former. In (c) and (d) multiple variably-sized spheroids were formed. All the images were taken 4 days post initiation. Scale bar: 200 μm. Click here for file

Additional file 3 **Glioblastoma spheroid-based migration on gelatin**. Day 4 U-87 MG or KNS42 tumor spheroids were transferred onto a gelatin-coated 96-well flat-bottomed plate (a single spheroid per well) and tumor cell dissemination was monitored. Selected images from a time lapse study illustrate fast, dispersed migration for U-87 MG and a slower radial, collective migration for KNS42. Images were obtained by microscopy as described in methods section. Scale bar: 200 μm. Click here for file

Additional file 4 **Video 1**. Glioblastoma spheroid-based migration on gelatin. The video shows side by side the differential migration patterns that characterize two glioblastoma cell lines (U-87 MG, adult and KNS42, pediatric). Images were obtained every 15 minutes over a period of 70 h, on an inverted microscope, Olympus IX 70, equipped with a Q Imaging CCD camera. Videos were edited using AVS Video Editor from Online Media Technologies Ltd. File format: MPEG. Click here for file

Additional file 5 **Video 2**. Tumor spheroid (TS)-embryoid body (EB) confrontation culture assay. Single day 4 U-87 MG TS and day 5 EBs were cocultured in each well of ultra-low attachment (ULA) 96-well plates. Timelapse imaging of the TS-EB confrontation culture shows the relatively rapid attachment and coalescence of the two tissues. Images were obtained on an inverted microscope every hour, over a period of 60 h. The video was edited as described above. File format: MPEG. Click here for file

Additional file 6 **Video 3**. Tumor spheroid (TS)-embryoid body (EB) confrontation culture assay. 17-*N*-Allylamino-17-demethoxygeldanamycin (17-AAG) inhibits U-87 MG tissue invasion. Single day 4 green fluorescent protein (GFP) transduced U-87 MG TS and day 7 EBs were cocultured in each well of ultra-low attachment (ULA) 96-well plates. Cocultures were treated with 17-AAG (5 μM) or with vehicle (control) at the initiation of the cocultures. Timelapse imaging shows the invasion of U-87 MG GFP cells towards the EB in the control coculture while no invasion is evident in the 17-AAG treated culture. Images were obtained as described above over a period of 40 h. File format: MPEG. Click here for file

Additional file 7 **Immunostaining of tumor spheroid (TS)-embryoid body (EB) at the end of a confrontation culture assay (60 h)**. Hematoxylin and eosin (H&E) staining (upper panel) shows the two tissues and their close association. CD34 immunoperoxidase staining (lower panel) identifies endothelial differentiation within the EB and the extension of the pseudovascular structures into the TS (arrows). Scale bars: 200 μm (left) and 100 μm (right) images. Click here for file

Additional file 8 **CellTiter Glo luminescent cell viability assay validation for three-dimensional cultures**. **(a) **CellTiter Glo assay of day 4 U-87 MG spheroids incubated for 10, 30 or 60 minutes with CellTiter Glo reagents showed close comparability of the luminescent signals. **(b) **Ultra-low attachment (ULA) plate generated U-87 MG spheroids were imaged and used in a CellTiter Glo luminescent assay on days 3, 4, 5, 6, 8 and 11 post initiation. Spheroid volumes (μm^3^) and luminescent counts showed a significant positive correlation (Spearman rank). **(c) **U-87 MG spheroids were initiated in ULA plates at different cell densities (5 × 10^4^, 2.5 × 10^4^, 12.5 × 10^4^, 6.25 × 10^4 ^and 3.12 × 10^4 ^cell/well). Then, 4 days later, spheroids were imaged and analyzed on a Celigo cytometer. Representative images of three of the cultures are shown. Scale bar: 500 μm. Following imaging, spheroids were subjected to a CellTiter Glo luminescent viability assay **(d)**. For assessment of the relationship between the number of viable cells and the luminescent signals from spheroids, a standard curve was generated. A single cell suspension of U-87 MG was plated into a 96-well plate, over a range of cell densities (10^5^, 10^4^, 10^3 ^and 10^2 ^cells/well) and immediately subjected to a CellTiter Glo assay alongside the spheroids **(e)**. Volumes (μm^3^) and the number of viable cells per spheroid determined using the standard curve showed a significant positive correlation (Spearman rank). Values are shown as means ± SD, n = 8. Click here for file

Additional file 9 **Scatter plots of the concentration inhibiting cell viability by 50% (GI_50_) values for cell viability studies summarized in Table **2. **(a-c) **U-87 MG, KNS42, LICR-LON-HN4 and MDA-MB-231 (P and M) cells were grown in two dimensions (monolayer) and in three dimensions (spheroids). Then, 4 days later, cells were treated with (a) 17-*N*-allylamino-17-demethoxygeldanamycin (17-AAG) (final concentrations 0 to 100 μM for U-87 MG and KNS42, 0 to 25 μM for MDA-MB-231 P and M, 0 to 1 μM for LICR-LON-HN4), (b) PI-103 (final concentrations 0 to 25 μM for U-87 MG and KNS42) and (c) CCT130234 (final concentrations 0 to 100 μM for U-87 MG and MDA-MB-231 P). Then, 72 h later, a CellTiter Glo luminescence assay for cell viability was performed and GI_50 _values were determined. Dots represent GI_50 _values from each assay and means ± SD are plotted. Click here for file

Additional file 10 **Comparison of sensitivity to compounds in two-dimensional vs three-dimensional cultures**. **(a, b) **MDA-MB-231 P cells were grown in two dimensions (monolayer) and in three dimensions (spheroids). Then, 4 days later, cells were treated with (a) 17-*N*-allylamino-17-demethoxygeldanamycin (17-AAG) (final concentrations 0 to 25 μM) or (b) CCT130234 (final concentrations 0 to 100 μM). Then, 72 h later, a CellTiter Glo luminescence assay for cell viability was performed and concentration inhibiting cell viability by 50% (GI_50_) values were determined. Values are means ± SD, n = 6; (a) and (b) are representative of three separate experiments. Click here for file

Additional file 11 **Volume distribution of control and treated U-87 MG spheroids**. Frequency plots of volume (μm^3 ^× 10^7^) distribution for controls (vehicle) and PI-103 treated (0.5 μM and 0.06 μM) U-87 MG spheroids on day 14 of a growth kinetic assay (n = 18). One example of three replicate studies (n = 6) is shown in Figure 6e. Click here for file

Additional file 12 **PI-103 and 17-*N*-allylamino-17-demethoxygeldanamycin (17-AAG) concentration-dependent inhibition of tumor spheroid growth kinetics: fully automated imaging and analysis**. **(a) **KNS42 and **(b) **LICR-LON-HN4 spheroids were treated on day 4 as described above, with PI-103 (0 to 2 μM) and 17-AAG (0 to 2.93 μM) respectively, accordingly to their three-dimensional concentration inhibiting cell viability by 50% (GI_50_) values (see Table 2). A Celigo cytometer was used for automated imaging and analysis. Graphs show concentration-dependent inhibition of spheroid growth from day 4 to day 14. Values are means ± SD, n = 6; one representative of three separate experiments is shown. Click here for file

Additional file 13 **17**-***N*-Allylamino-17-demethoxygeldanamycin (17-AAG) concentration-dependent inhibition of tumor spheroid growth kinetics: alternative non-automated analysis**. **(a) **U-87 MG and **(b) **LICR-LON-HN4 spheroids were treated on day 4 with 17-AAG (0 to 2 μM for U-87 MG and 0 to 2.93 μM for LICR-LON-HN4), according to their three-dimensional concentration inhibiting cell viability by 50% (GI_50_) values (see Table 2). Images were obtained by microscopy as described above and analysis of spheroid growth was performed using Image-Pro Analyzer software. Graphs show a concentration-dependent growth inhibition of the spheroids from day 4 to day 14. Values are means ± SD, n = 6; one representative of three separate experiments is shown for each cell line. Click here for file

Additional file 14 **17-*N*-Allylamino-17-demethoxygeldanamycin (17-AAG) mediated concentration-dependent inhibition of tumor spheroid invasion into Matrigel**. **(a-d) **day 4 U-87 MG or MDA-MB-231 M (metastatic variant) spheroids were embedded into Matrigel for three-dimensional invasion. 17-AAG was used over a range of concentrations and control spheroids were treated with vehicle. Images were captured at intervals from t0 to 72 h using a Celigo cytometer for U-87 MG or an inverted microscope for MDA-MB-231 M. The area of invasion was determined using Image-Pro Analyzer software. Representative examples of three separate experiments are shown for U-87 MG (a, b) and for MDA-MB-231 M (c, d). Values are means ± SD, n = 6. (b, d) Representative images of spheroid invasion were obtained on a Celigo cytometer (b) or by microscopy (d) at t0 and 24 h. Scale bar: 500 μm. Click here for file

Additional file 15 **Imaging parameters: Celigo cytometer and microscopy**. Technical details. Click here for file
