# Supplementary figures and images for: Advances in establishment and analysis of three-dimensional tumor spheroid-based functional assays for target validation and drug evaluation
Source: BMC Biol. 2012 Mar 22;10:29. doi: 10.1186/1741-7007-10-29 (PMC3349530; doi:10.1186/1741-7007-10-29)

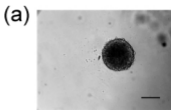

SF188  
Tight spheroid

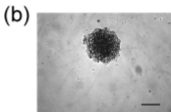

MDA MB 231  
Compact aggregate

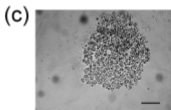

IGROV-1  
Loose aggregate

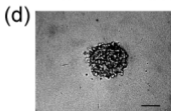

Compact aggregate  
(+ matrigel™)

Supplement: Additional file 1 — Classification of three-dimensional tumor spheroid morphology. Representative examples are shown: (a) SF188: tight spheroid; (b) MDA-MB-231 P: compact aggregate; (c) IGROV-1: loose aggregate; (d) IGROV-1 compact aggregate upon Matrigel addition. Scale bar: 200 μm. [file 1741-7007-10-29-S1.PDF]

# U-87 MG

(a)

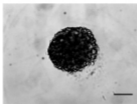

ULA

(b)

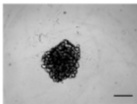

agar

(c)

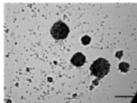

poly-Hema

(d)

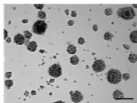

RCCS

Supplement: Additional file 2 — Generation of tumor spheroids: comparative methods. U-87 MG spheroids were generated in (a) U-bottomed 96-well ultra-low attachment (ULA) plates, (b) flat-bottomed 96-well agar-coated plates, (c) 24-well poly-Hema-coated plates and (d) in a Rotary Cell Culture System (RCCS; Cellon). In (a) and (b) one spheroid/well was obtained with a tighter structure in the former. In (c) and (d) multiple variably-sized spheroids were formed. All the images were taken 4 days post initiation. Scale bar: 200 μm. [file 1741-7007-10-29-S2.PDF]

U-87 MG

KNS42

t0

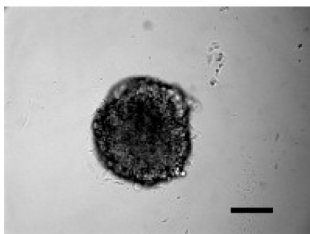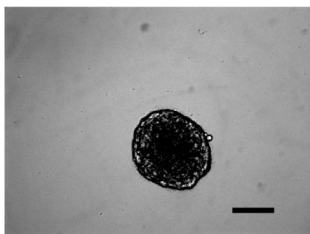

6 h

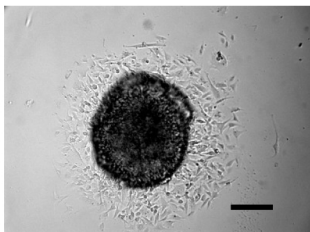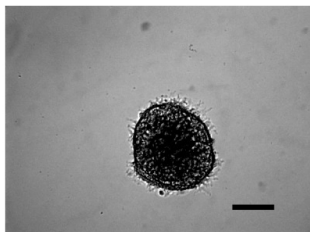

12.5 h

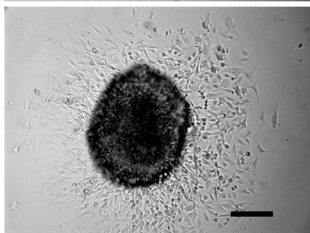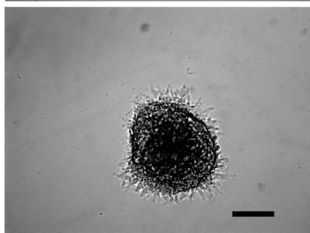

25 h

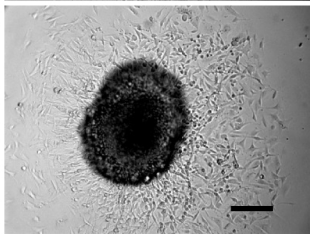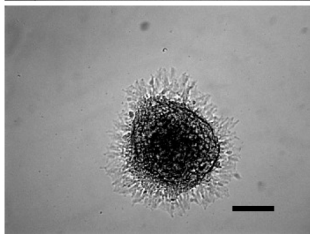

50 h

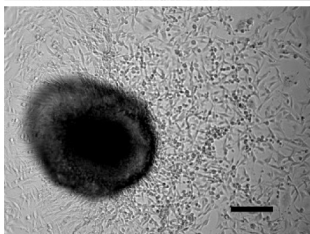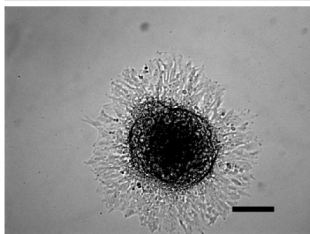

70 h

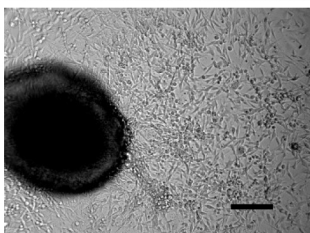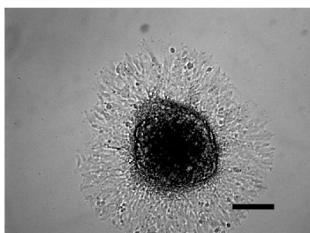

Supplement: Additional file 3 — Glioblastoma spheroid-based migration on gelatin. Day 4 U-87 MG or KNS42 tumor spheroids were transferred onto a gelatin-coated 96-well flat-bottomed plate (a single spheroid per well) and tumor cell dissemination was monitored. Selected images from a time lapse study illustrate fast, dispersed migration for U-87 MG and a slower radial, collective migration for KNS42. Images were obtained by microscopy as described in methods section. Scale bar: 200 μm. [file 1741-7007-10-29-S3.PDF]

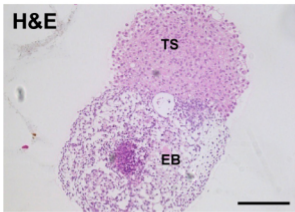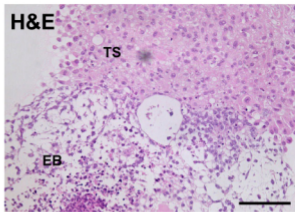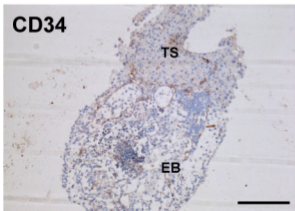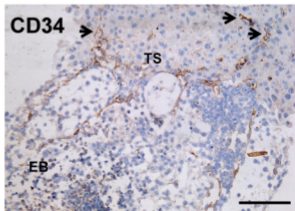

Supplement: Additional file 7 — Immunostaining of tumor spheroid (TS)-embryoid body (EB) at the end of a confrontation culture assay (60 h). Hematoxylin and eosin (H&E) staining (upper panel) shows the two tissues and their close association. CD34 immunoperoxidase staining (lower panel) identifies endothelial differentiation within the EB and the extension of the pseudovascular structures into the TS (arrows). Scale bars: 200 μm (left) and 100 μm (right) images. [file 1741-7007-10-29-S7.PDF]

(a)

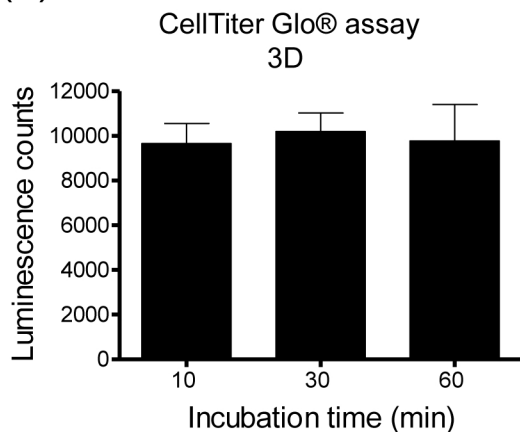

(b)

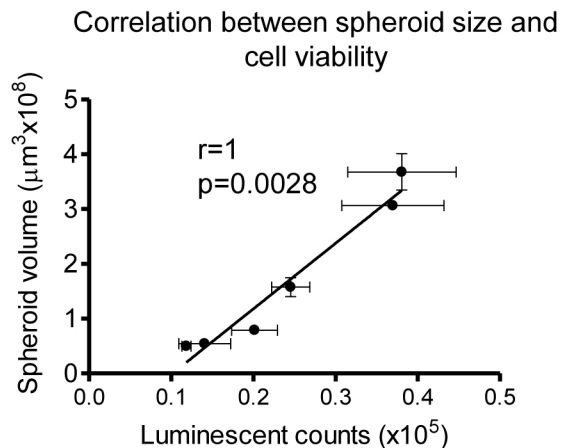

(c)

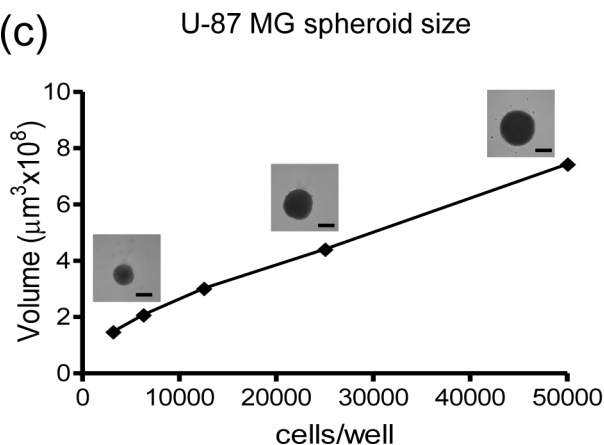

(d)

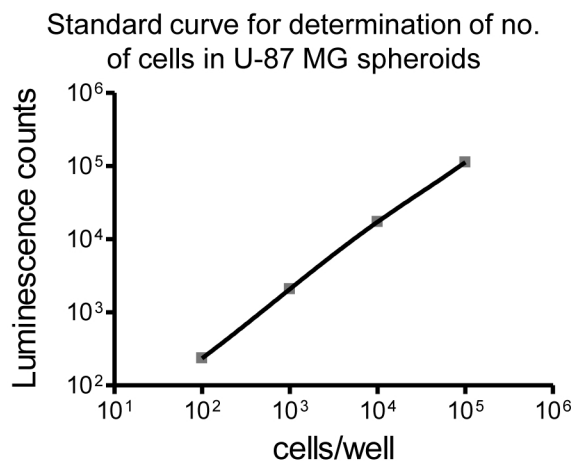

(e)

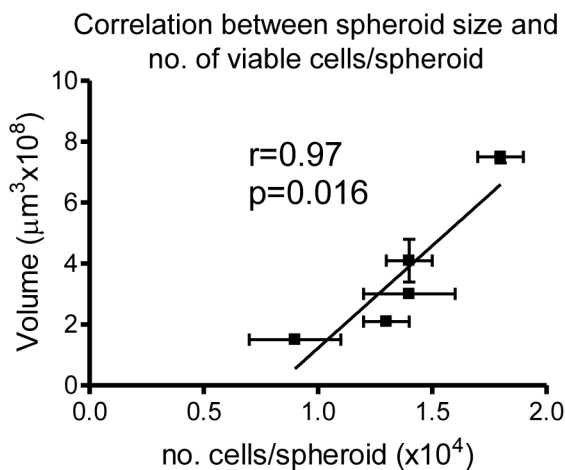

Supplement: Additional file 8 — CellTiter Glo luminescent cell viability assay validation for three-dimensional cultures. (a) CellTiter Glo assay of day 4 U-87 MG spheroids incubated for 10, 30 or 60 minutes with CellTiter Glo reagents showed close comparability of the luminescent signals. (b) Ultra-low attachment (ULA) plate generated U-87 MG spheroids were imaged and used in a CellTiter Glo luminescent assay on days 3, 4, 5, 6, 8 and 11 post initiation. Spheroid volumes (μm3) and luminescent counts showed a significant positive correlation (Spearman rank). (c) U-87 MG spheroids were initiated in ULA plates at different cell densities (5 × 10^4^, 2.5 × 10^4^, 12.5 × 104, 6.25 × 10^4^ and 3.12 × 10^4^ cell/well). Then, 4 days later, spheroids were imaged and analyzed on a Celigo cytometer. Representative images of three of the cultures are shown. Scale bar: 500 μm. Following imaging, spheroids were subjected to a CellTiter Glo luminescent viability assay (d). For assessment of the relationship between the number of viable cells and the luminescent signals from spheroids, a standard curve was generated. A single cell suspension of U-87 MG was plated into a 96-well plate, over a range of cell densities (105, 104, 103 and 102 cells/well) and immediately subjected to a CellTiter Glo assay alongside the spheroids (e). Volumes (μm3) and the number of viable cells per spheroid determined using the standard curve showed a significant positive correlation (Spearman rank). Values are shown as means ± SD, n = 8. [file 1741-7007-10-29-S8.PDF]

**A**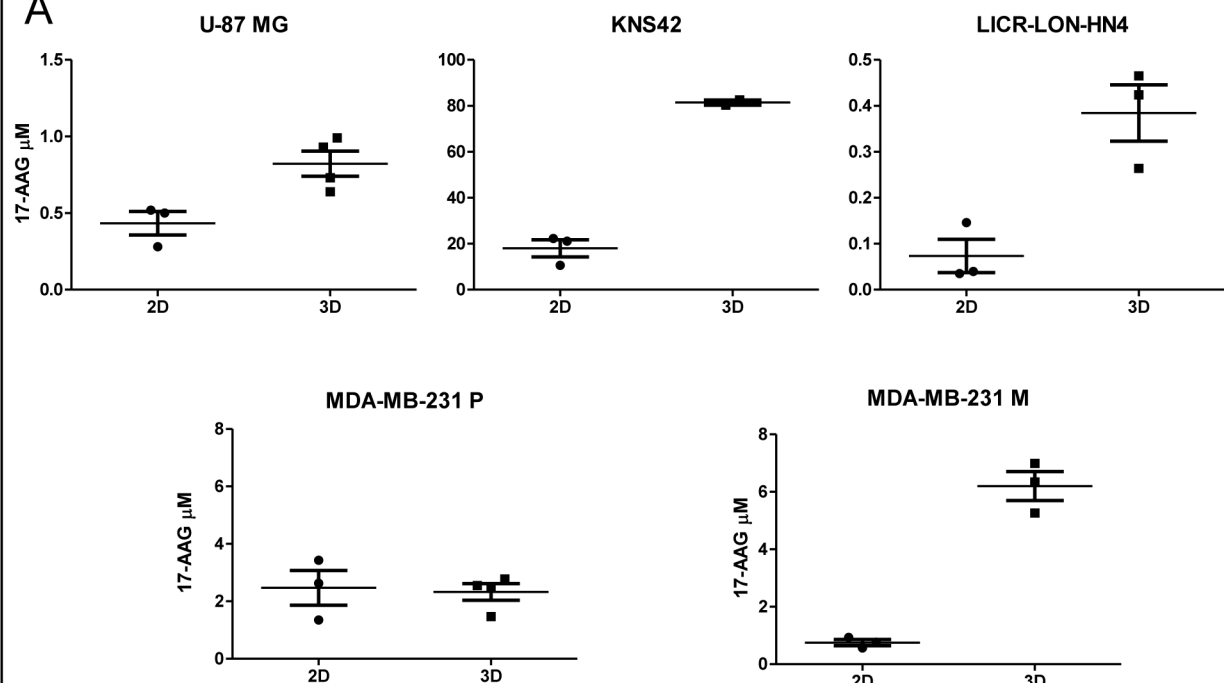**B**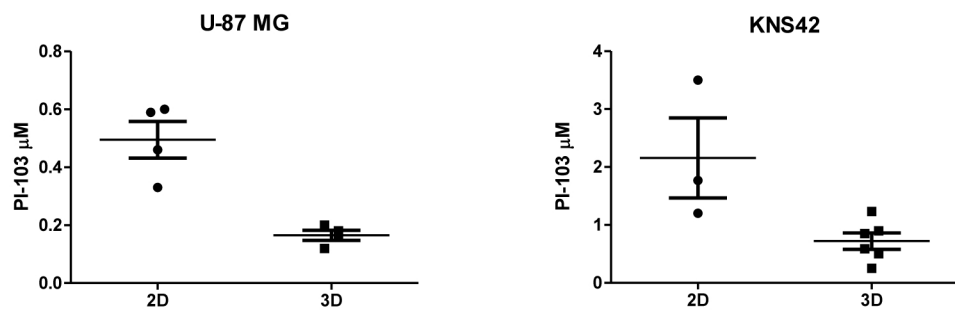**C**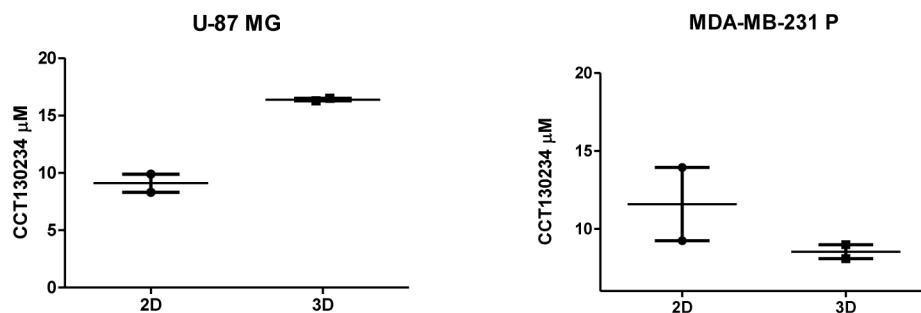

Supplement: Additional file 9 — Scatter plots of the concentration inhibiting cell viability by 50% (GI50) values for cell viability studies summarized in Table 2. (a-c) U-87 MG, KNS42, LICR-LON-HN4 and MDA-MB-231 (P and M) cells were grown in two dimensions (monolayer) and in three dimensions (spheroids). Then, 4 days later, cells were treated with (a) 17-N-allylamino-17-demethoxygeldanamycin (17-AAG) (final concentrations 0 to 100 μM for U-87 MG and KNS42, 0 to 25 μM for MDA-MB-231 P and M, 0 to 1 μM for LICR-LON-HN4), (b) PI-103 (final concentrations 0 to 25 μM for U-87 MG and KNS42) and (c) CCT130234 (final concentrations 0 to 100 μM for U-87 MG and MDA-MB-231 P). Then, 72 h later, a CellTiter Glo luminescence assay for cell viability was performed and GI50 values were determined. Dots represent GI50 values from each assay and means ± SD are plotted. [file 1741-7007-10-29-S9.PDF]

(a)

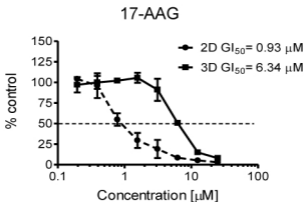

(b)

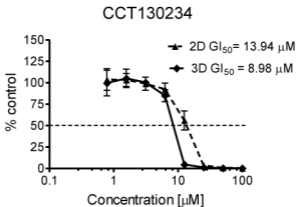

Supplement: Additional file 10 — Comparison of sensitivity to compounds in two-dimensional vs three-dimensional cultures. (a, b) MDA-MB-231 P cells were grown in two dimensions (monolayer) and in three dimensions (spheroids). Then, 4 days later, cells were treated with (a) 17-N-allylamino-17-demethoxygeldanamycin (17-AAG) (final concentrations 0 to 25 μM) or (b) CCT130234 (final concentrations 0 to 100 μM). Then, 72 h later, a CellTiter Glo luminescence assay for cell viability was performed and concentration inhibiting cell viability by 50% (GI50) values were determined. Values are means ± SD, n = 6; (a) and (b) are representative of three separate experiments. [file 1741-7007-10-29-S10.PDF]

U-87 MG spheroids treated with PI-103  
growth kinetic day 14

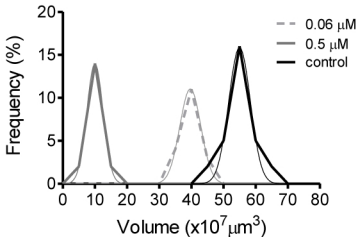

Supplement: Additional file 11 — Volume distribution of control and treated U-87 MG spheroids. Frequency plots of volume (μm3 × 107) distribution for controls (vehicle) and PI-103 treated (0.5 μM and 0.06 μM) U-87 MG spheroids on day 14 of a growth kinetic assay (n = 18). One example of three replicate studies (n = 6) is shown in Figure 6e. [file 1741-7007-10-29-S11.PDF]

(a)

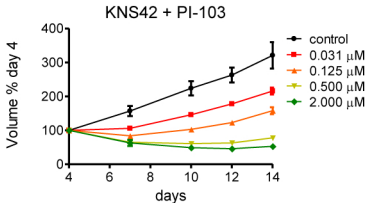

(b)

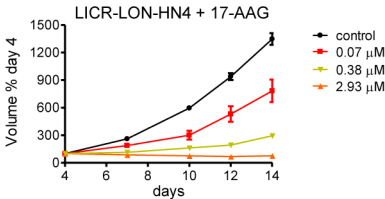

Supplement: Additional file 12 — PI-103 and 17-N-allylamino-17-demethoxygeldanamycin (17-AAG) concentration-dependent inhibition of tumor spheroid growth kinetics: fully automated imaging and analysis. (a) KNS42 and (b) LICR-LON-HN4 spheroids were treated on day 4 as described above, with PI-103 (0 to 2 μM) and 17-AAG (0 to 2.93 μM) respectively, accordingly to their three-dimensional concentration inhibiting cell viability by 50% (GI50) values (see Table 2). A Celigo cytometer was used for automated imaging and analysis. Graphs show concentration-dependent inhibition of spheroid growth from day 4 to day 14. Values are means ± SD, n = 6; one representative of three separate experiments is shown. [file 1741-7007-10-29-S12.PDF]

(a)

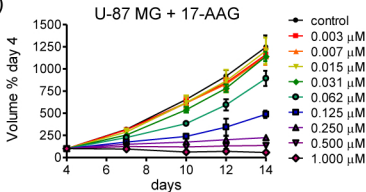

(b)

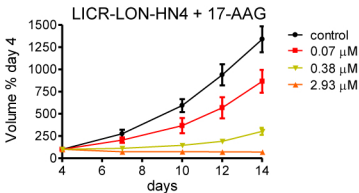

Supplement: Additional file 13 — 17-N-Allylamino-17-demethoxygeldanamycin (17-AAG) concentration-dependent inhibition of tumor spheroid growth kinetics: alternative non-automated analysis. (a) U-87 MG and (b) LICR-LON-HN4 spheroids were treated on day 4 with 17-AAG (0 to 2 μM for U-87 MG and 0 to 2.93 μM for LICR-LON-HN4), according to their three-dimensional concentration inhibiting cell viability by 50% (GI50) values (see Table 2). Images were obtained by microscopy as described above and analysis of spheroid growth was performed using Image-Pro Analyzer software. Graphs show a concentration-dependent growth inhibition of the spheroids from day 4 to day 14. Values are means ± SD, n = 6; one representative of three separate experiments is shown for each cell line. [file 1741-7007-10-29-S13.PDF]

(a)

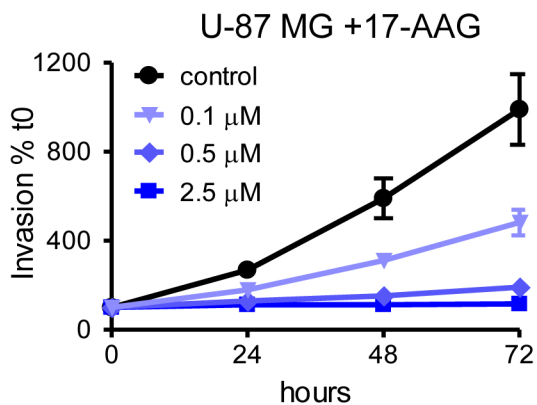

(b)

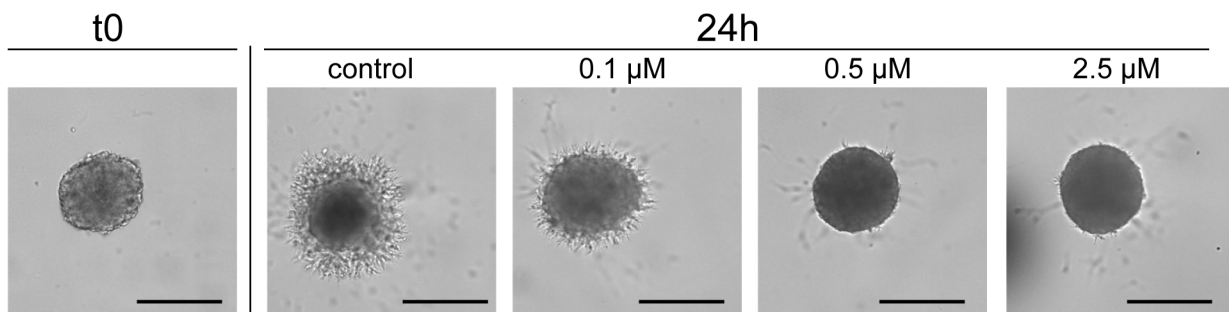

(c)

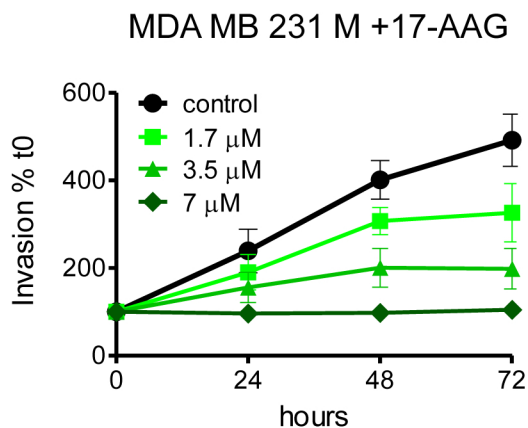

(d)

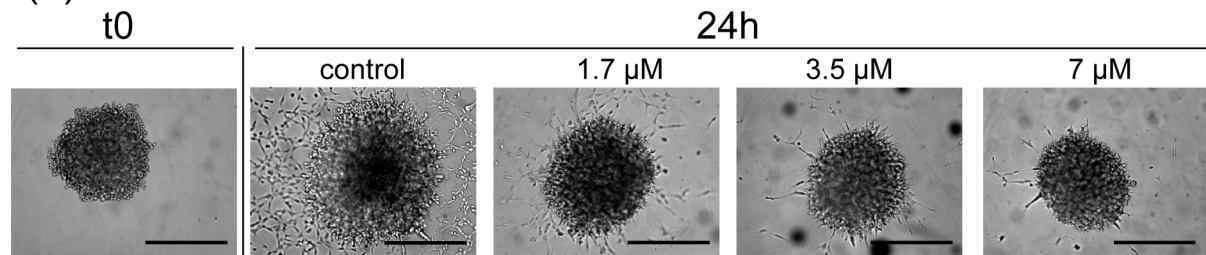

Supplement: Additional file 14 — 17-N-Allylamino-17-demethoxygeldanamycin (17-AAG) mediated concentration-dependent inhibition of tumor spheroid invasion into Matrigel. (a-d) day 4 U-87 MG or MDA-MB-231 M (metastatic variant) spheroids were embedded into Matrigel for three-dimensional invasion. 17-AAG was used over a range of concentrations and control spheroids were treated with vehicle. Images were captured at intervals from t0 to 72 h using a Celigo cytometer for U-87 MG or an inverted microscope for MDA-MB-231 M. The area of invasion was determined using Image-Pro Analyzer software. Representative examples of three separate experiments are shown for U-87 MG (a, b) and for MDA-MB-231 M (c, d). Values are means ± SD, n = 6. (b, d) Representative images of spheroid invasion were obtained on a Celigo cytometer (b) or by microscopy (d) at t0 and 24 h. Scale bar: 500 μm. [file 1741-7007-10-29-S14.PDF]
