# Supplementary material for: Advances in establishment and analysis of three-dimensional tumor spheroid-based functional assays for target validation and drug evaluation
Source: BMC Biol. 2012 Mar 22;10:29. doi: 10.1186/1741-7007-10-29 (PMC3349530; doi:10.1186/1741-7007-10-29)
Supplement: Additional file 15 — Imaging parameters: Celigo cytometer and microscopy. Technical details. [file 1741-7007-10-29-S15.DOC]

|  | μm/pixel | image dimension (pixels)  width x height | image dimension (μm)  width x height | File size |
| --- | --- | --- | --- | --- |
| Celigo™ cytometer   - EB application - Confluence application | 2.11  1.06 | 975 x 976  1958 x 1958 | 2057 x 2060  2055 x 2055 | 0.41 MB  1.55 MB |
| Microscope   - 10x objective - 4x objective | 1.0  2.5 | 1392 x 1040  1392 x 1040 | 1392 x 1040  3513 x 2625 | 2.18 MB  2.18 MB |

**Additional file 15**

Imaging technical information
